# Uncovering the anticancer mechanism of Compound Kushen Injection against HCC by integrating quantitative analysis, network analysis and experimental validation

**DOI:** 10.1038/s41598-017-18325-7

**Published:** 2018-01-12

**Authors:** Li Gao, Ke-xin Wang, Yu-zhi Zhou, Jian-song Fang, Xue-mei Qin, Guan-hua Du

**Affiliations:** 1 Modern Research Center for Traditional Chinese Medicine, Shanxi University, Taiyuan, 030006 PR China; 2 College of Chemistry and Chemical Engineering, Shanxi University, Taiyuan, 030006 China; 3 Institute of Clinical Pharmacology, Guangzhou University of Chinese Medicine, Guangzhou, 510405 China; 4 Institute of Materia Medica, Chinese Academy of Medical Sciences & Peking Union Medical College, Beijing, 100050 China

## Abstract

Compound Kushen Injection (CKI) is a Traditional Chinese Medicine (TCM) preparation that has been clinically used in China to treat various types of solid tumours. Although several studies have revealed that CKI can inhibit the proliferation of hepatocellular carcinoma (HCC) cell lines, the active compounds, potential targets and pathways involved in these effects have not been systematically investigated. Here, we proposed a novel idea of “main active compound-based network pharmacology” to explore the anti-cancer mechanism of CKI. Our results showed that CKI significantly suppressed the proliferation and migration of SMMC-7721 cells. Four main active compounds of CKI (matrine, oxymatrine, sophoridine and N-methylcytisine) were confirmed by the integration of ultra-performance liquid chromatography/mass spectrometry (UPLC-MS) with cell proliferation assays. The potential targets and pathways involved in the anti-HCC effects of CKI were predicted by a network pharmacology approach, and some of the crucial proteins and pathways were further validated by western blotting and metabolomics approaches. Our results indicated that CKI exerted anti-HCC effects via the key targets MMP2, MYC, CASP3, and REG1A and the key pathways of glycometabolism and amino acid metabolism. These results provide insights into the mechanism of CKI by combining quantitative analysis of components, network pharmacology and experimental validation.

## Introduction

Hepatocellular carcinoma (HCC) is the 3rd leading cause of cancer-related death worldwide, and its incidence is increasing^1^. Approximately three-quarters of HCC cases are attributed to chronic HBV and HCV infections^2^. In recent years, substantial evidence has shown that genetic alterations^3,4^ and metabolic disorders^5,6^ also play critical roles in the pathogenesis of HCC. Most HCC patients are diagnosed at an advanced stage with few therapeutic measures. Trans-arterial chemoembolization (TACE), radiotherapy and chemotherapy are the current treatment modalities for HCC, and sorafenib is the only drug that has been approved by the FDA^7^.

Compound Kushen Injection (CKI) is derived from two herbs, *Radix sophorae flavescentis* and *Rhizoma smilacis glabrae*. CKI has been clinically used in China for over 15 years for the treatment of many types of solid tumours, especially for cancer-related pains^8^. CKI combined with TACE treatment could elevate the therapeutic efficacy of unresectable HCC^9^. CKI may deliver anti-HCC effects through multiple compounds acting on multiple targets and pathways. Qu *et al*. used functional genomics to identify the anti-cancer mechanisms of CKI in the MCF-7 cell line. They found that CKI exerted anti-cancer effects likely through the regulation of the cell cycle, cell apoptosis, lncRNAs and other pathways^10^. However, the candidate mechanisms underlying the anti-HCC effects of CKI are still unknown.

Network pharmacology, first proposed by Andrew L Hopkins^11^, has greatly promoted the mechanistic study of Traditional Chinese Medicine^12–14^. This approach has advantages in interpreting the synergistic effects of Traditional Chinese Medicine (TCM) with multiple components and multiple targets. In most studies, network pharmacology considers drug-like ingredients in herb databases, while the contents of the ingredients are often neglected. Thus, the primary ingredients and targets predicted by network pharmacology may deviate from the truth.

**Figure 1:**
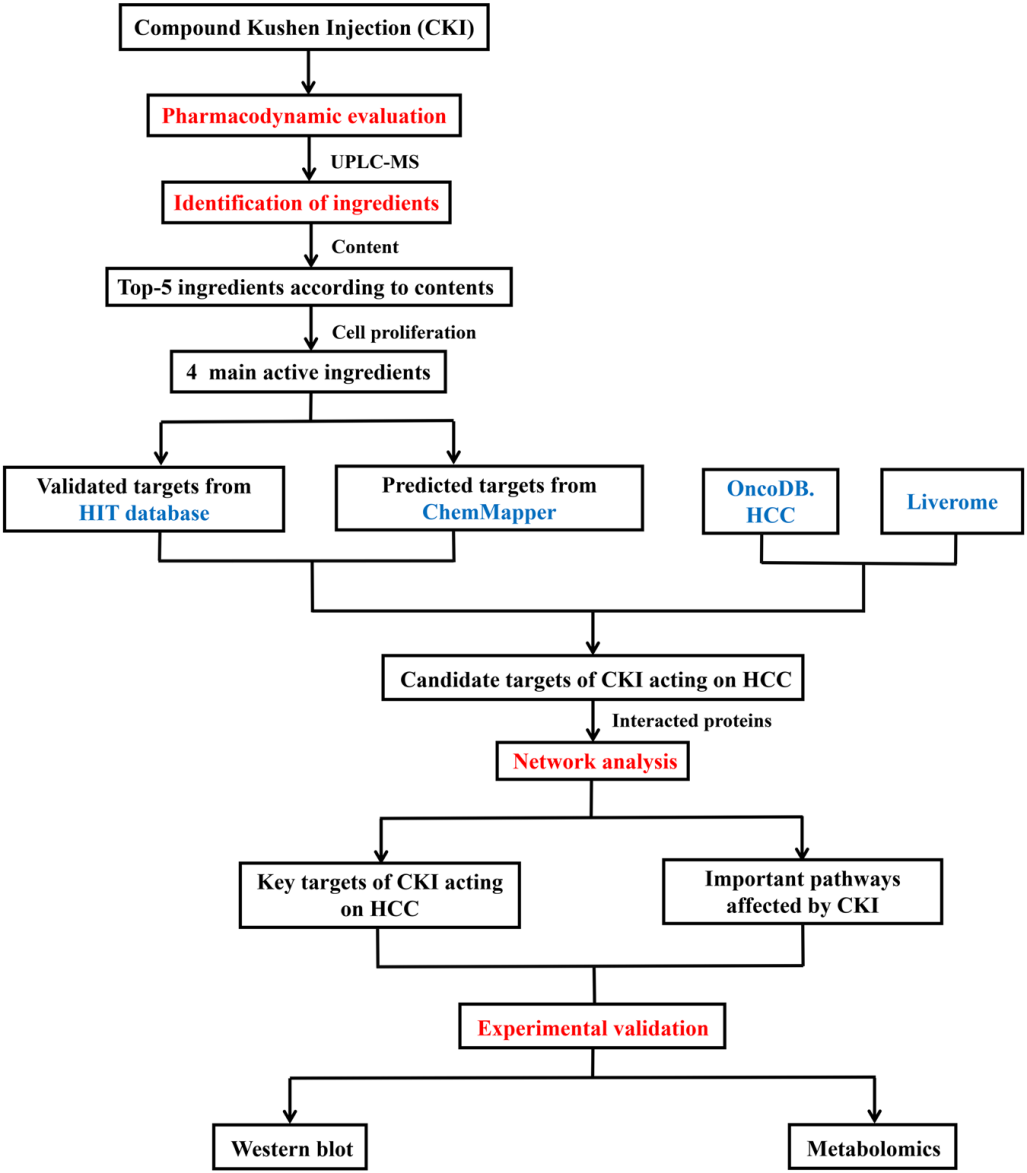
A schematic diagram of the integrative strategy combining quantitative analysis of components, network analysis and experimental validation for investigation of the mechanisms of Compound Kushen Injection (CKI) against HCC.

In the current study, network pharmacology analysis was performed focusing on the main active compounds of CKI. The workflow is illustrated in Fig. 1 as follows: (1) the anti-HCC effects of CKI were evaluated in SMMC-7721 cells; (2) the ingredients of CKI were quantitatively analysed by UPLC-MS; (3) the effects of the primary compounds (top 5 in content) of CKI on cell proliferation were measured to identify the main active compounds; (4) the 4 main active compounds were used for network pharmacology analysis to predict the potential targets and pathways of CKI against HCC; (5) the key targets and pathway were validated by experiments.

## Results

### CKI inhibits the proliferation and migration of SMMC-7721 cells

**Figure 2:**
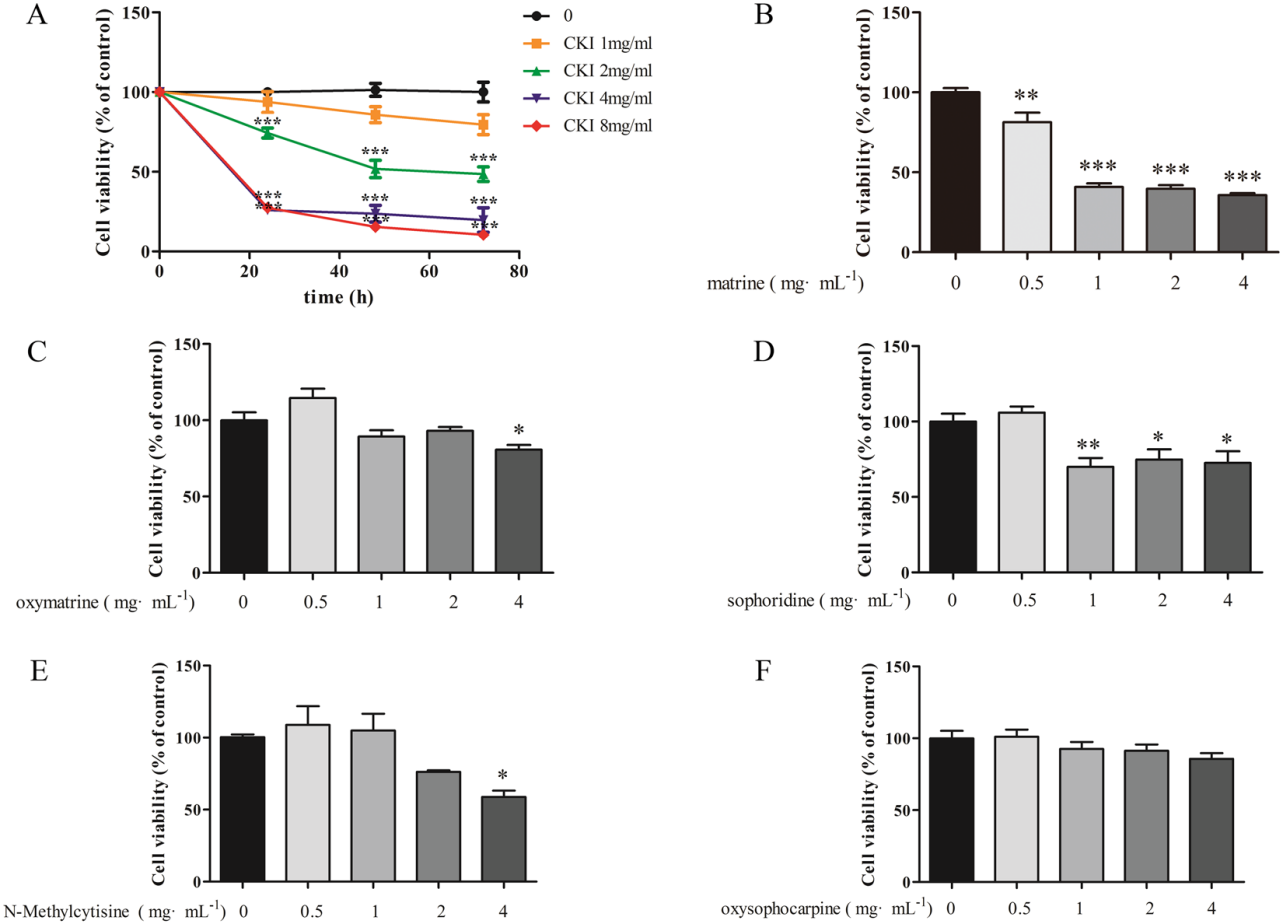
Effects of CKI and its components on proliferation of SMMC-7721 cells. (**A**) CKI, (**B**) matrine, (**C**) oxymatrine, (**D**) sophoridine, (**E**) N-methylcytisine, (**F**) oxysophocarpine. The cell viabilities under different treatments were measured using MTT. Data are represented as mean ± SEM (n = 6). *p < 0.05, **p < 0.01, ***p < 0.001 versus control group.

**Figure 3:**
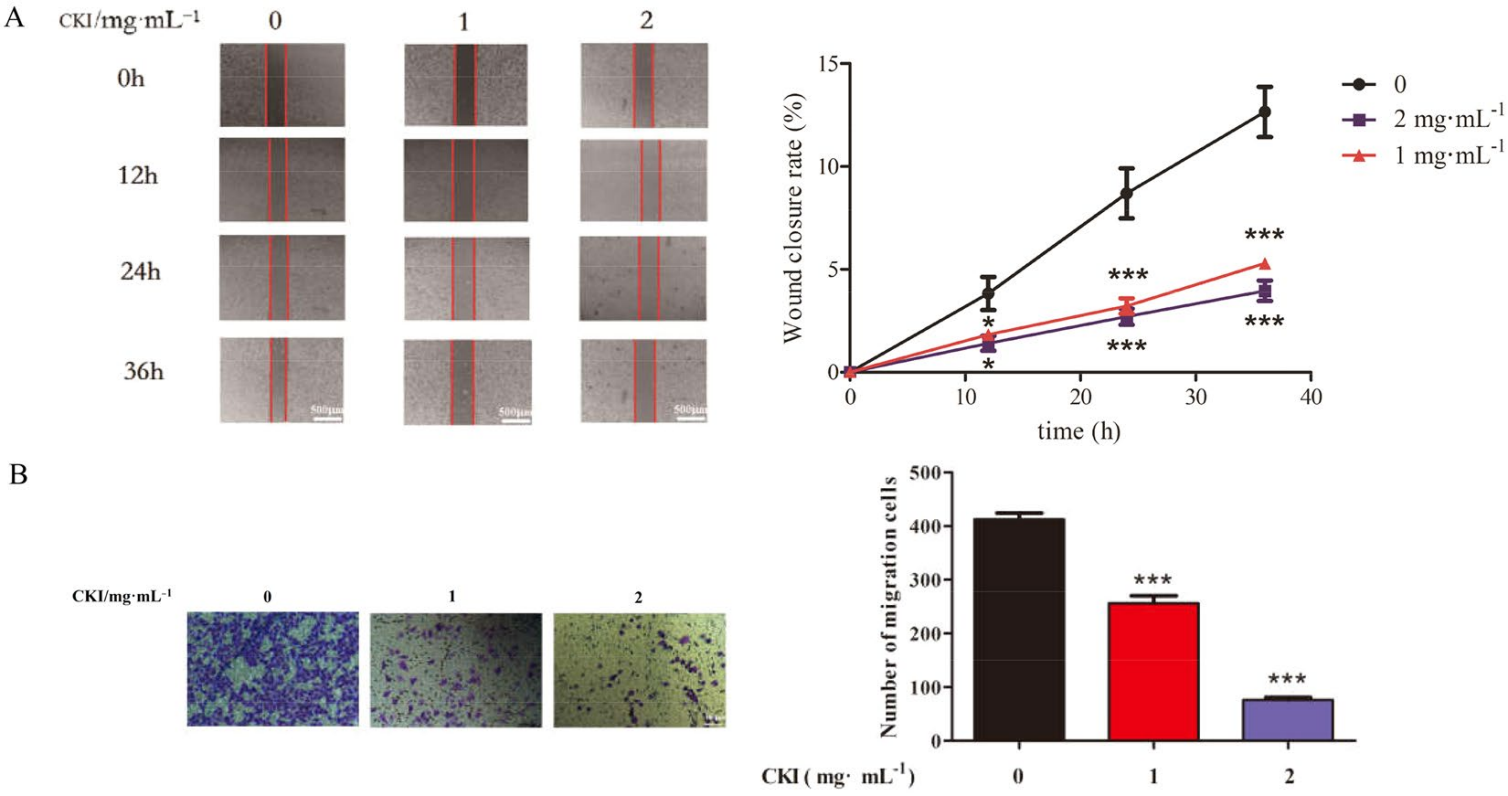
CKI can inhibit the migration of SMMC-7721 cells. (**A**) Wound-healing assays show that CKI (1 and 2 mg/mL) significantly inhibits the migration of SMMC-7721 cells at 12, 24 and 36 h. (**B**) Transwell migration assays show that CKI (1 and 2 mg/mL) significantly inhibits the migration of SMMC-7721 cells at 36 h. *p < 0.05, ***p < 0.001 versus untreated cells.

The effects of CKI on the proliferation and migration of SMMC**-**7721 cells were determined. MTT assays showed that 2, 4 and 8 mg/mL CKI dramatically inhibited the proliferation of SMMC-7721 cells at 24, 48, and 72 h in a time-dependent manner (Fig. 2A). Wound-healing and transwell assays showed that 1 and 2 mg/mL significantly suppressed the migration of SMMC**-**7721 cells (Fig. 3). These results suggest that CKI displayed significant inhibitory activities on the proliferation and migration of SMMC**-**7721 cells.

### Identification of chemical ingredients in CKI by UPLC-MS

**Figure 4:**
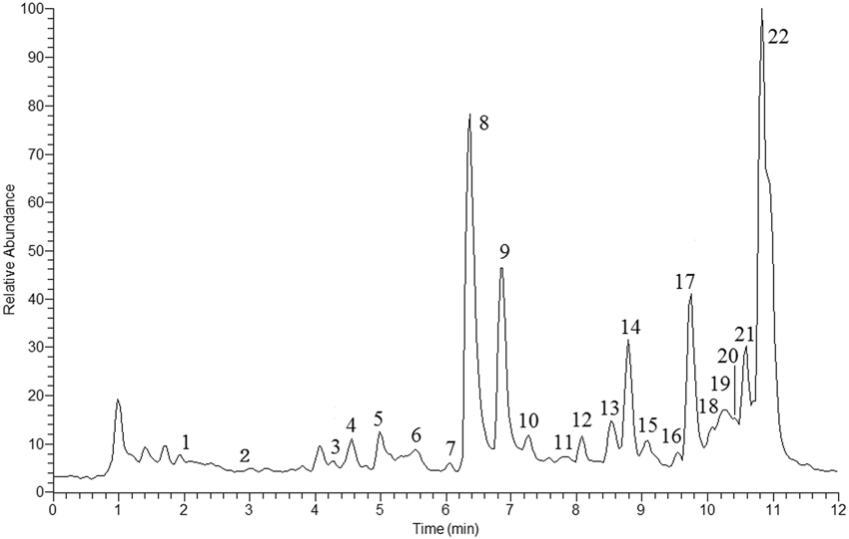
Positive total ion current chromatography (TIC) of CKI.

UPLC**-**MS is a rapid, reliable and accurate technique to identify the chemical ingredients in TCM. In the present study, 22 ingredients of CKI were identified by UPLC-MS (Fig. 4, Table 1). The top five compounds by content were matrine (6.07 mg/mL), oxymatrine (5.46 mg/mL), oxysophocarpine (3.89 mg/mL), sophoridine (2.23 mg/mL) and N-methylcytisine (1.14 mg/mL). These 5 compounds were further identified by comparing the retention times and accurate masses with those of standard substances (Supplementary Figure S1–S5). Other compounds were determined by comparing the retention time and mass spectra with those of authentic substances and the reported data in the literature.

**Table 1:** Identified Compounds in CKI by UPLC-MS.

No.	t_R_(min)	compounds	Molecular formula	[M + H]^+^	Fragment ions
1	2.28	oxymamanine	C_15_H_23_O_3_N_2_	279.1702	177.1386, 261.1595, 557.3329
2	2.54	9β-hydroxylamprolobine N-oxide	C_15_H_25_O_4_N_2_	297.1808	279.1700, 593.3537
3	4.29	9β-hydroxylamprolobine	C_15_H_25_O_3_N_2_	281.1859	148.1120, 263.1751, 561.3642
4	4.56	5α, 9α-hydroxymatrine	C_15_H_25_O_3_N_2_	281.1859	148.1114, 245.1646, 263.1750, 561.3648
5	4.99	9α -hydroxymatrine	C_15_H_25_O_2_N_2_	265.1910	177.1379, 247.1801, 529.3748
6	5.52	oxysophoranol	C_15_H_25_O_3_N_2_	281.1859	148.1121, 245.1645, 263.1753, 561.3643
7	6.05	no identified		283.2015	152.1433
8	6.22	oxymatrine	C_15_H_25_O_2_N_2_	265.1910	148.1119, 150.1275, 247.1801 [M + H_2_O]^+^, 529.3748 [2M + H]^+^
9	6.53	oxysophocarpine	C_15_H_23_O_2_N_2_	263.1753	150.1275, 245.1647 [M + H_2_O]^+^, 525.3426 [2M + H]^+^
10	7.27	mamanine	C_15_H_23_O_2_N_2_	263.1753	150.1275, 231.1496, 245.1645 [M + H_2_O]^+^, 525.3434 [2M + H]^+^
11	7.82	sophoranol	C_15_H_25_O_2_N_2_	265.1910	148.1117, 150.1279, 247.1802 [M + H_2_O]^+^
12	8.09	oxysophoridine	C_15_H_25_O_2_N_2_	265.1909	164.1607, 247.1808 [M + H_2_O]^+^
13	8.52	baptifoline	C_15_H_21_O_2_N_2_	261.1598	114.0915,243.1488
14	8.25	N-methylcyticine	C_12_H_17_ON_2_	205.1335	146.0601
15	9.09	9α-hydroxysophocarpine	C_15_H_23_O_3_N_2_	279.1703	261.1595 [M + H_2_O]^+^
16	9.58	(-)-oxylehmannine	C_15_H_23_O_2_N_2_	263.1754	245.1647
17	9.47	sophoridine	C_15_H_25_ON_2_	249.1960	150.1280
18	10.12	14β-hydrsophoridine	C_15_H_25_O_2_N_2_	265.1910	247.1805
19	10.22	(-)-acetylbaptifoline	C_17_H_24_O_3_N_2_	303.1702	243.1491
20	10.39	Sophocarpine	C_15_H_22_ON_2_	247.1805	148.1121, 150.1278
21	10.59	isomatrine	C_15_H_25_ON_2_	249.1961	150.1279
22	10.82	matrine	C_15_H_25_ON_2_	249.1960	148.1120, 150.6241, 134.5847, 231.1852 [M + H_2_O]^+^

### Identification of the main active compounds in CKI

MTT assay was used to screen the active compounds against HCC from the top-5 compounds. Our results showed that matrine, oxymatrine, sophoridine and N**-**methylcytisine, each at 4 mg/mL significantly inhibited the proliferation of SMMC**-**7721 cells at 24 h (Fig. 2B–E). These 4 compounds were considered the main active compounds of CKI. However, even at 4 mg/ml, oxysophocarpine showed no obvious effect on the proliferation of SMMC-7721 cells after 24 h of treatment (Fig. 2F).

### Network construction of the anti-HCC targets of CKI

**Figure 5:**
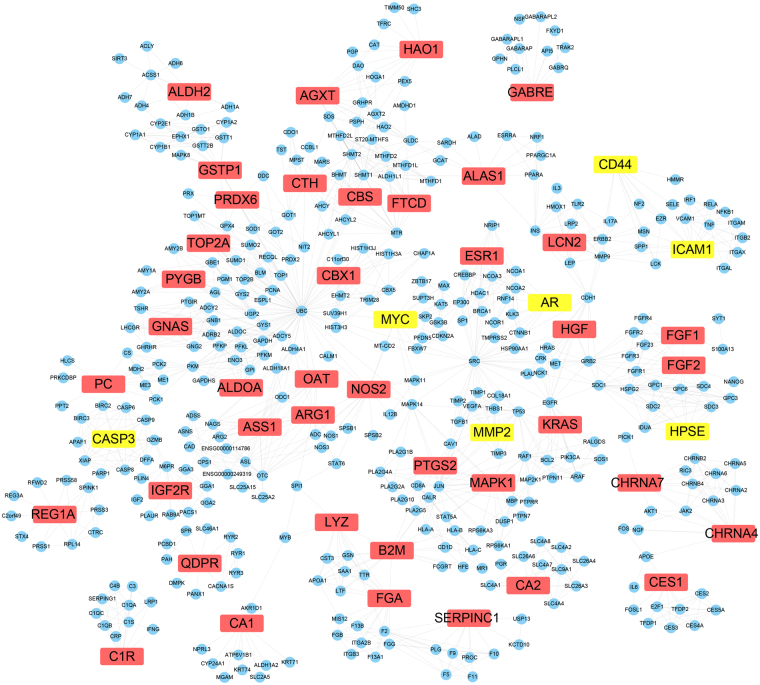
The network of CKI targets-interacted proteins. The yellow nodes represent validated targets, the red nodes represent predicted targets and the blue nodes represent associated proteins of targets.

In our previous study^13^, 566 genes that were significant to HCC were collected from the OncoDB.HCC^15^ and Liverome databases^16^. The validated targets and predicted targets of the main active compounds of CKI were collected and then mapped to these genes to obtain shared genes, which were predicted to be the candidate targets of CKI. Forty-eight targets of CKI were obtained, including 7 validated targets (AR, CASP3, CD44, HPSE, ICAM**-**1, MMP2, and MYC) and 41 predicted targets; 384 proteins associated with the 48 targets of CKI were acquired from the STRING database. The protein**-**protein interaction network of CKI for the anti**-**HCC effects consisted of 432 proteins through 1540 interactions with an average degree of 7.13 (Fig. 5).

### Network topological analysis

NetworkAnalyzer was used to calculate the average shortest path lengths and betweenness centrality of the 48 targets of CKI, and the results are shown in Table 2. The topological parameters such as shortest path length and betweenness centrality were usually used for the analysis of key nodes in the network. Nodes with a small average shortest path length and large betweenness centrality value were considered as important proteins in the network^17^. The R value was used to evaluate the importance of the 48 targets as equation (1). From the predicted targets of the main active compounds, CASP3, MYC, and MMP2 were predicted as crucial targets of CKI from the validated targets, and QDPR, GABRE, and REG1A were predicted as crucial targets of CKI.

**Table 2:** 48 targets of CKI with average shortest path length and betweenness centrality.

Swiss prot	Genes/proteins	Description	validated or predicted	Average shortest path length	Betweenness centrality	R
P09417	QDPR	quinoid dihydropteridine reductase	predicted	1.00	0.80000	0.0000
P78334	GABRE	Gamma-aminobutyric-acid receptor subunit epsilon precursor	predicted	1.00	0.73889	0.0000
P05451	REG1A	Lithostathine 1 alpha precursor	predicted	1.00	0.70370	0.0000
P00918	CA2	carbonic anhydrase II	predicted	1.00	0.26688	0.0000
P00736	C1R	complement component 1, r subcomponent	predicted	1.00	0.14741	0.0000
P11717	IGF2R	insulin-like growth factor 2 receptor	predicted	1.65	0.33684	0.0613
P43681	CHRNA4	Neuronal acetylcholine receptor subunit alpha-4	predicted	1.79	0.51600	0.0746
P42574	CASP3	Caspase-3	validated	1.90	0.04377	0.0849
P23141	CES1	carboxylesterase 1	predicted	2.13	0.45833	0.1060
P36544	CHRNA7	Neuronal acetylcholine receptor subunit alpha-7	predicted	2.29	0.09934	0.1217
P35228	NOS2	Nitric oxide synthase	predicted	2.98	0.05616	0.1865
Q92597	MYC	Myc proto-oncogene protein	validated	3.07	0.04787	0.1951
P05089	ARG1	Arginase-1	predicted	3.23	0.10864	0.2099
P00966	ASS1	argininosuccinate synthase 1	predicted	3.37	0.03375	0.2231
P09211	GSTP1	Glutathione S-transferase P	predicted	3.45	0.10380	0.2251
P04181	OAT	Ornithine aminotransferase	predicted	3.38	0.00546	0.2261
P32929	CTH	cystathionase	predicted	3.40	0.01890	0.2279
P35520	CBS	Cystathionine beta-synthase	predicted	3.41	0.00541	0.2310
P08253	MMP2	Matrix metalloproteinase-2	validated	3.46	0.00064	0.2341
P14210	HGF	Hepatocyte growth factor precursor	predicted	3.47	0.00046	0.2373
P30041	PRDX6	peroxiredoxin 6	predicted	3.48	0.00605	0.2400
P04075	ALDOA	aldolase A	predicted	3.52	0.00072	0.2403
P11216	PYGB	phosphorylase, glycogen; brain	predicted	3.54	0.00500	0.2407
Q5JWF2	GNAS	Guanine nucleotide-binding protein G(s) subunit alpha isoforms XLas	predicted	3.54	0.00253	0.2418
P83916	CBX1	chromobox homolog 1	predicted	3.55	0.00130	0.2425
P11388	TOP2A	topoisomerase (DNA) II alpha 170 kDa	predicted	3.55	0.00104	0.2432
P28482	MAPK1	Mitogen-activated protein kinase 1	predicted	3.64	0.02154	0.2486
P03372	ESR1	Estrogen receptor	predicted	3.63	0.00146	0.2503
P10275	AR	Androgen receptor	validated	3.65	0.00819	0.2505
P35354	PTGS2	Prostaglandin G/H synthase 2 precursor	predicted	3.67	0.00602	0.2524
P61769	B2M	beta-2-microglobulin	predicted	3.97	0.11074	0.2751
P13196	ALAS1	aminolevulinate, delta-, synthase 1	predicted	3.92	0.03940	0.2796
P01116	KRAS	GTPase KRas	predicted	4.15	0.00012	0.2983
P80188	LCN2	Neutrophil gelatinase-associated lipocalin precursor	predicted	4.16	0.00751	0.2985
P21549	AGXT	alanine-glyoxylate aminotransferase	predicted	4.17	0.02412	0.3024
O95954	FTCD	formiminotransferase cyclodeaminase	predicted	4.20	0.00607	0.3059
P61626	LYZ	lysozyme	predicted	4.25	0.07924	0.3166
P09038	FGF2	Heparin-binding growth factor 2 precursor	predicted	4.35	0.00683	0.3184
P05230	FGF1	Heparin-binding growth factor 1 precursor	predicted	4.38	0.01212	0.3249
Q9Y251	HPSE	Heparanase precursor	validated	4.39	0.00001	0.3266
P11498	PC	pyruvate carboxylase	predicted	4.45	0.01814	0.3524
P02671	FGA	fibrinogen alpha chain	predicted	4.74	0.04801	0.3790
P05362	ICAM1	Intercellular adhesion molecule 1 precursor	validated	5.02	0.05416	0.3813
P16070	CD44	CD44 antigen precursor	validated	5.04	0.00918	0.3886
Q9UJM8	HAO1	hydroxyacid oxidase (glycolate oxidase) 1	predicted	5.12	0.01655	0.4330
P00915	CA1	Carbonic anhydrase 1	predicted	5.59	0.05388	0.4817
P01008	SERPINC1	serpin peptidase inhibitor, clade C (antithrombin), member 1	predicted	6.11	0.01214	0.5011
P05091	ALDH2	aldehyde dehydrogenase 2 family (mitochondrial)	predicted	6.31	0.00301	0.8195

### Pathway enrichment analysis

Reactome was used to explore the potential pathways affected by CKI through analysis of the 48 targets. The pathways were ranked by their nominal p values with a cut**-**off of 0.001 (Table 3). Seven pathways were enriched: Metabolism of amino acids and derivatives, Metabolism, FRS2**-**mediated cascade, FGFR1b ligand binding and activation, Amyloids, Reversible hydration of carbon dioxide and SHC**-**mediated cascade.

**Table 3:** Pathway enrichment for the targets of CKI.

Reactome Pathway	P-value	FDR	HitGenes
Metabolism of amino acids and derivatives	0	0.002252408	AGXT, QDPR, CTH, OAT, ASS1, ARG1, FTCD, CBS
Metabolism	0.0001	0.019183984	PTGS2, AGXT, ALAS1, CD44, HPSE, QDPR, CTH, GNAS, CA2, CA1, OAT, GSTP1
FRS2-mediated cascade	0.0002	0.026828354	FGF1, FGF2, MAPK1, KRAS
FGFR1b ligand binding and activation	0.0005	0.046185577	FGF1, FGF2
Amyloids	0.0007	0.046185577	B2M, LYZ, FGA
Reversible hydration of carbon dioxide	0.0008	0.046185577	CA2, CA1
SHC-mediated cascade	0.001	0.046185577	FGF1, FGF2, KRAS

### Experimental validation of key targets

**Figure 6:**
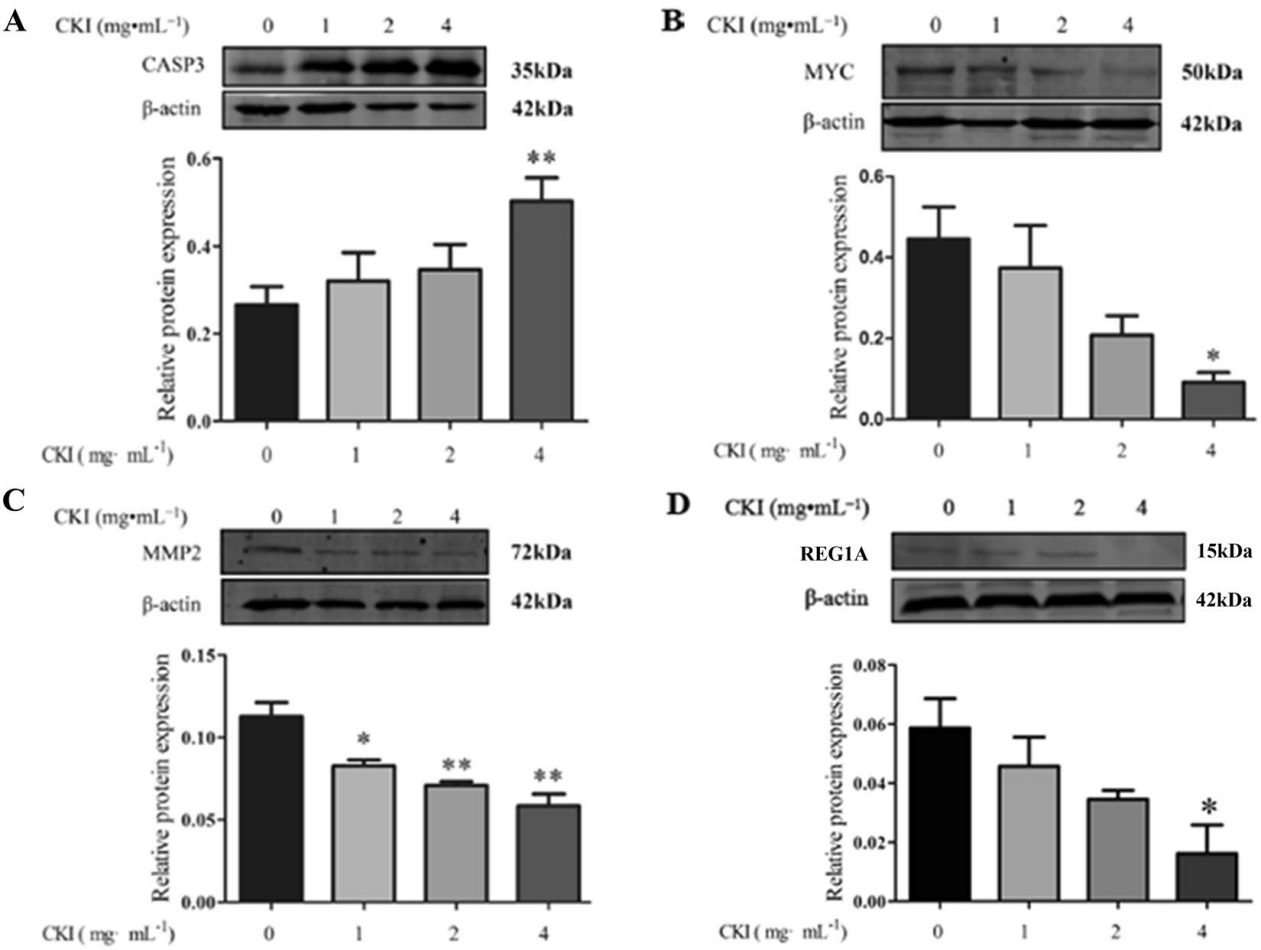
The effects of CKI on protein levels of CASP3 (**A**), MYC (**B**), MMP2 (**C**) and REG1A (**D**) in SMMC-7721 cells. β-Actin was used as loading control. n = 3, $\overline{x}$ ± SEM. *p < 0.05, **p < 0.01 versus untreated cells.

To delineate the anti**-**HCC mechanisms of CKI, some of the crucial proteins predicted by network pharmacology were experimentally validated in SMMC**-**7721 cells in response to CKI treatment. As shown in Fig. 6, CKI significantly inhibited the expression of MMP2, MYC, and REG1A and significantly increased the expression of caspase 3 in a dose**-**dependent manner.

### Experimental validation of the metabolism pathway

Metabolomics was used to detect the metabolism pathways affected by CKI. The typical ^1^H**-**NMR spectra of cell and medium are present in Supplementary Figure S6. Resonance assignments (Supplementary Table S1) were performed based on the chemical shifts of standard compounds from the Chenomx NMR suite, the Human Metabolome Database (HMDB)^18^, and Biological Magnetic Resonance Data Bank (BMRB)^19^, as well as the literature data^20,21^. The NMR spectra of cell and medium were dominated by peaks from amino acids, organic acids, choline-containing metabolites, and amine metabolites, in addition to some other metabolites.

**Figure 7:**
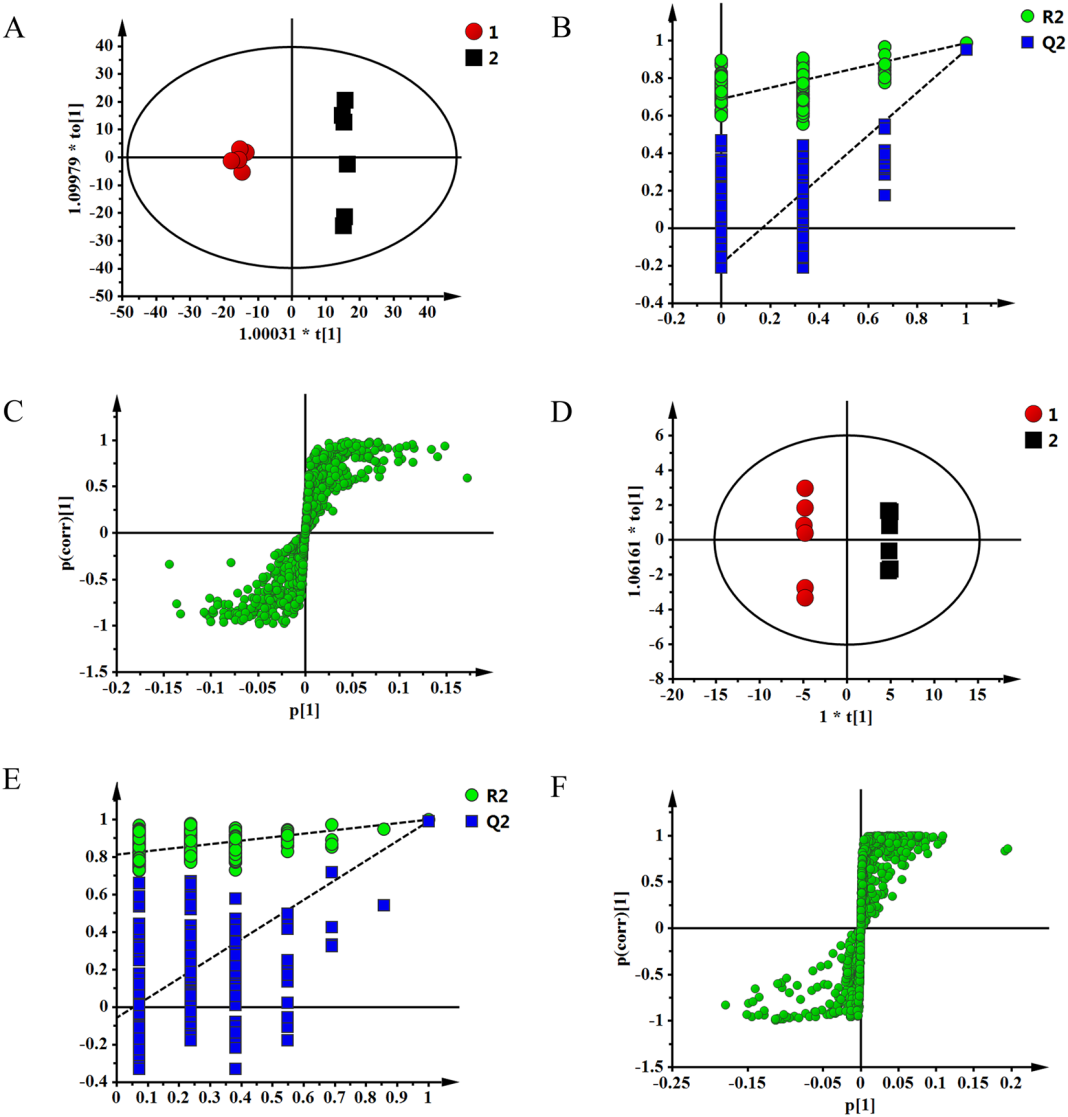
OPLS scores plot (**A**), corresponding validation plot (**B**) and S-plot (**C**) derived from SMMC-7721 cells; OPLS scores plot (**D**), corresponding validation plot (**E**) and S-plot (**F**) derived from medium.

To obtain more details of the metabolic differences after treatment with CKI, all the NMR data were subjected to multivariate data analysis. A partial least squares discriminant analysis (PLS-DA) model was further constructed and validated using the response of the permutation test through 200 permutations in which all R^2^ and Q^2^ values were lower than the original ones deemed to be of great predictive ability and reliability. The good PLS-DA models (cell model parameters: R^2^X = 0.632, Q^2^ = 0.979; medium model parameters: R^2^X = 0.741, Q^2^ = 0.993) indicate excellent predictive powers. Potential biomarkers associated with CKI treatment were further identified by OPLS-DA. The corresponding S-plot, VIP values and t-tests were used to test the statistical significance of the altered metabolites and to find metabolites contributing to the separation (Fig. 7). Compared with the control group, 16 differential metabolites were confirmed in cells, including higher levels of leucine, valine, acetate, glutamine, glycerol, β-glucose, tyrosine and phenylalanine and lower levels of glutamate, glutathione, creatine, GPC^c^, glycine, 1,3-dihydroxyacetone, adenosine monophosphate and hypoxanthine. In the medium 10 differential metabolites were confirmed, including higher levels of pyruvate, succinate, pyroglutamate, glycine, threonine, glycerol and alanine and lower levels of leucine, valine and lactate. CKI could significantly regulate the contents of different metabolites and attenuate the metabolic disorders in hepatoma cells (Table 4).

**Table 4:** The differential metabolites in SMMC-7721 cell and medium after treatment of CKI. *p < 0.05, **p < 0.01, ***p < 0.001 versus untreated cells.

No	Metabolites	CKI cell	fold change	CKI medium	fold change
1	Leucine	↑**	4.00	↓***	1.97
2	Valine	↑**	5.39	↓***	1.60
3	Acetate	↑***	4.81		
4	Lactate			↓***	5.25
5	Pyruvate			↑***	4.91
6	Succinate			↑***	2.17
7	Pyroglutamate			↑***	2.29
8	Glutamate	↓***	2.98		
9	Glutamine	↑**	1.52		
10	Glutathione	↓***	4.10		
11	Creatine	↓**	2.61		
12	GPC^c^	↓**	2.57		
13	Glycine	↓***	2.91	↑***	1.67
14	Threonine			↑***	12.76
15	1,3-Dihydroxyacetone	↓**	2.01		
16	Glycerol	↑***	2.31	↑**	2.08
17	Alanine			↑***	1.32
18	Adenosine monophosphate	↓*	1.52		
19	β-Glucose	↑**	11.05		
20	Tyrosine	↑***	3.69		
21	Phenylalanine	↑***	3.34		
22	Hypoxanthine	↓***	3.18		

**Figure 8:**
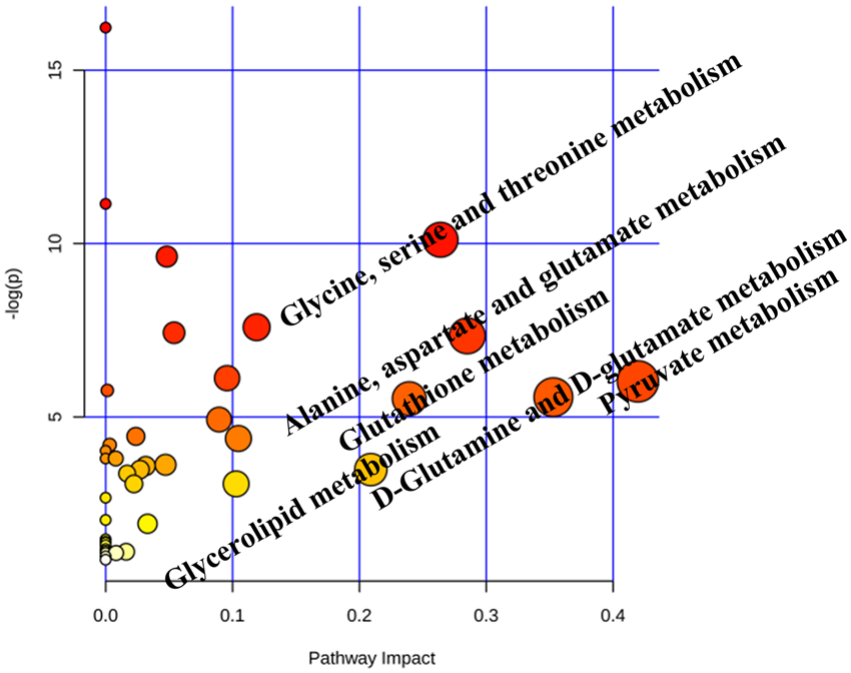
MetPA analysis of metabolic pathway.

**Figure 9:**
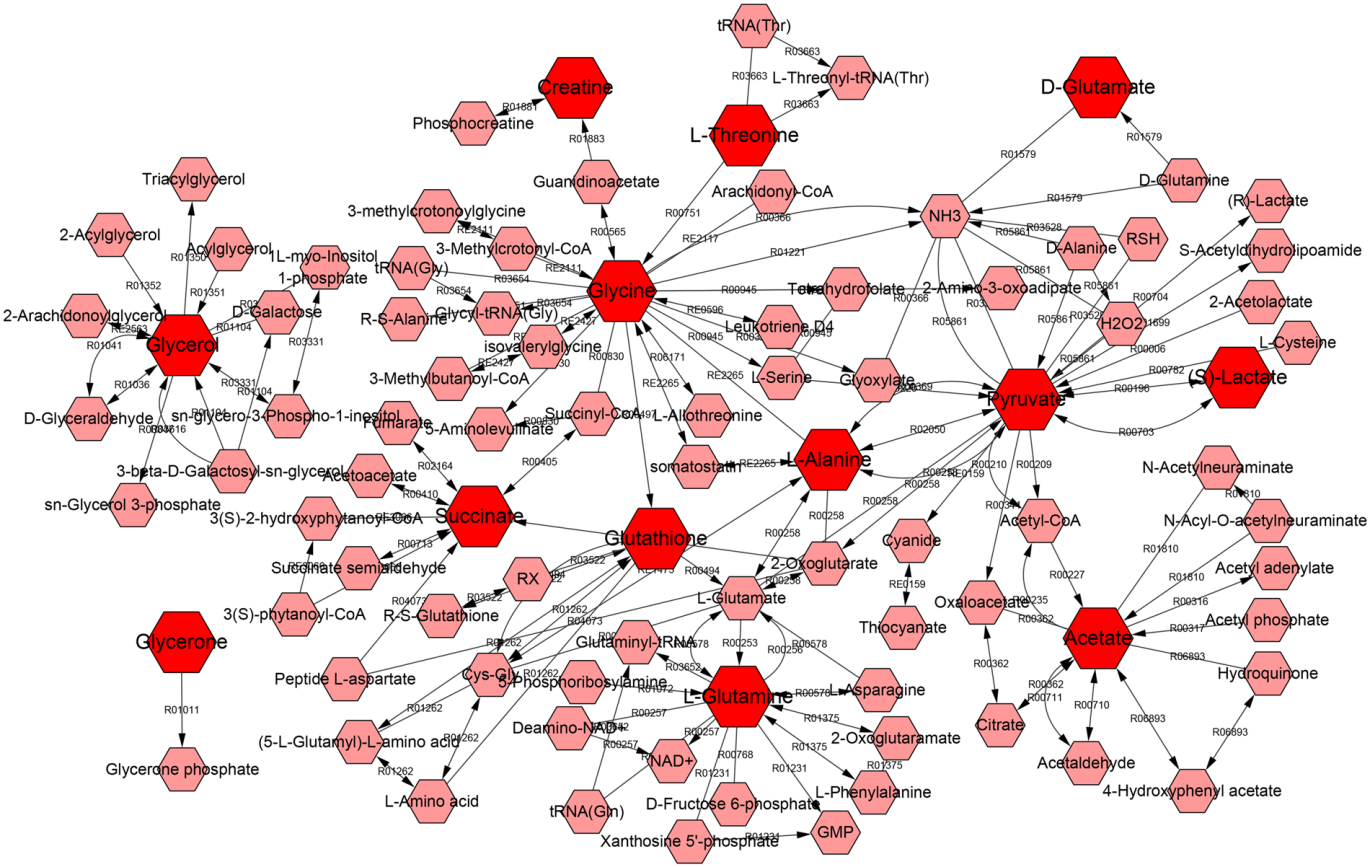
The network of potential biomarkers of CKI for the anti-HCC effect. The figure was constructed using MetScape and the nodes represent metabolites and edges represent biochemical reactions.

Metabolites with significant abundance changes between the CKI treatment and control groups were subjected to pathway analysis using MetaboAnalyst 3.0 and the KEGG database (www.genome.jp/kegg/) (Fig. 8). The most relevant metabolic pathways regulated by CKI included Pyruvate metabolism; D-Glutamine and D-glutamate metabolism; Glycine, serine and threonine metabolism; Alanine, aspartate and glutamate metabolism; Glutathione metabolism; and Glycerolipid metabolism. To gain additional insights into the relationship between metabolites, the differential biomarkers were mapped to KEGG IDs and the metabolite network was constructed by MetScape (Fig. 9).

### Effects of CKI on the levels of pyruvate and glutamate

**Figure 10:**
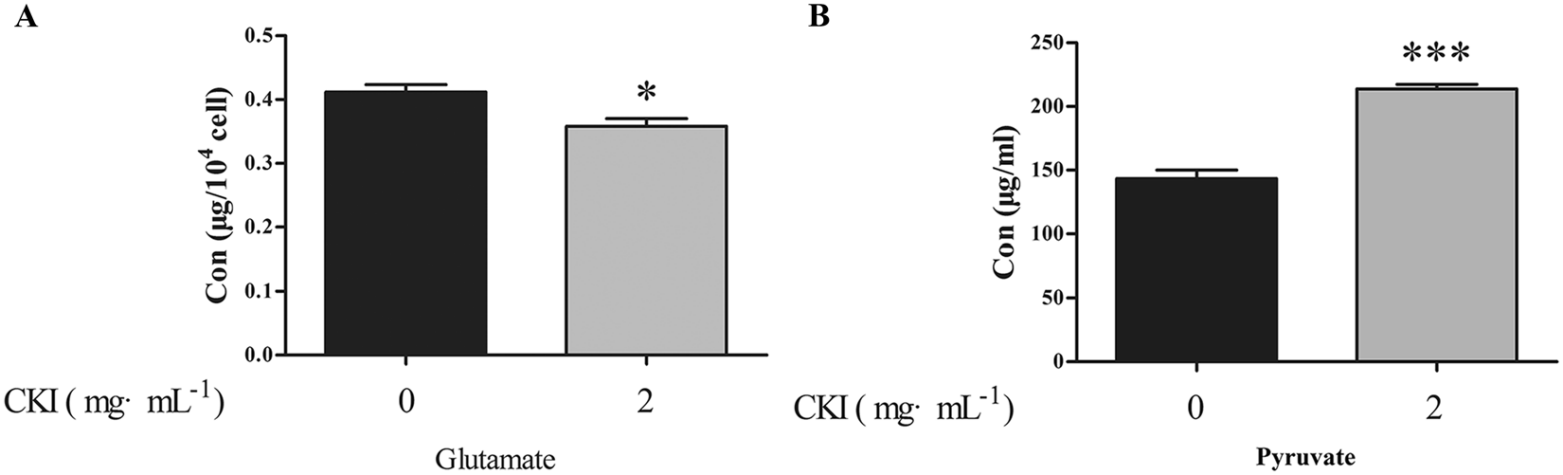
Effects of CKI on the contents of glutamate (**A**) and pyruvate (**B**) of SMMC-7721 cells. n = 3, $\overline{x}$ ± SEM. *p < 0.05, ***p < 0.001 versus untreated cells.

Pyruvate participates in pyruvate metabolism; glycine, serine and threonine metabolism; alanine, aspartate and glutamate metabolism. Glutamate is involved in D**-**Glutamine and D**-**glutamate metabolism. Thus, representative metabolites pyruvate and glutamate were selected to identify the variation between the CKI treatment and control groups. CKI significantly increased the content of pyruvate in the medium but decreased the content of glutamate in the cell (Fig. 10).

### Compound-target-metabolite network of CKI

**Figure 11:**
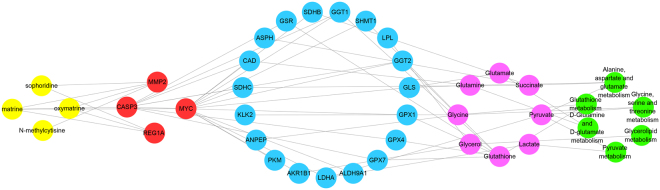
Compound-target-metabolite network. The nodes of active compounds, targets, pathway genes, differential metabolites, and metabolism pathway were colored in yellow, red, blue, purple, and green, respectively.

The compound-target-metabolite network was constructed for the 4 main active compounds and 4 experimentally validated targets. As shown in Fig. 11, matrine acted on the key targets of CASP3, MMP2, and MYC; oxymatrine acted on the key targets of MMP2 and REG1A; sophoridine acted on the key targets of MMP2 and REG1A; and N-methylcytisine acted on the key targets of MMP2. By acting on these targets and interacting targets, CKI could regulate the metabolites and metabolic pathways.

## Discussion

CKI has been approved by the State Food and Drug Administration of China for over 15 years and is widely known for its pain relief roles in cancer. Increasing evidence has shown that CKI combined with radiotherapy significantly improved the clinical efficacies of acute leukaemia^22^, non-small cell lung cancer^23^, and HCC^9^. Particularly, the combination treatment of TACE and CKI can improve the 1-and 2-year survival rates in patients with unresectable HCC. Our results suggested that CKI significantly suppressed the proliferation and migration of SMMC-7721 cells. However, the mechanisms of anti-HCC effects of CKI needed further investigation.

Network pharmacology embraces some aspects of biological networks, such as connectivity, redundancy and pleiotropy^24^, allowing it to provide insights into biological systems^25^. Network pharmacology is a useful approach to investigate the mechanisms of TCM. Generally, in previous studies, all ingredients of herbs were collected from herb databases, such as TCMSP^26^, TCM database@ Taiwan^27^, and TCMID^28^. In some cases, the ingredients were filtered according to ADMET properties or drug-likeness value^13,29^. However, few studies have focused on the main active compounds of TCM via network pharmacology analysis.

Here, we proposed a novel idea of “main active compound-based network pharmacology”. The concept was consistent with the method proposed by Li. *et al*.^30,31^, which was based on the combination of chemical and therapeutic properties with network pharmacology. Li’s approach used computational methods to predict the role and mechanism of herbal formulae. The method could easily integrate newly found ingredients and then provide a more comprehensive understanding of the herbal formula. However, in our approach, network pharmacology analysis was performed based on the main active compounds, which filters out some ineffective compounds and focuses on the high content and active compounds. Our approach provides a novel strategy to achieve an accurate and systematic exploration of the mechanisms of TCM.

In this study, 22 compounds of CKI were identified by UPLC-MS, and the top 5 compounds by content (matrine, oxymatrine, sophoridine, N-methylcytisine and oxysophocarpine) were identified by comparison with standard substances. Subsequent assays showed that 4 compounds (matrine, oxymatrine, sophoridine, and N-methylcytisine) at the maximum of 4 mg/kg could markedly inhibit the proliferation of SMMC-7721 cells. These 4 compounds were considered main active compounds of CKI. The mechanisms of the 4 main active compounds predicted by network pharmacology were speculated to be the mechanisms of CKI.

By integration of the validated targets and predicted targets of the 4 compounds, the key targets of CKI were predicted according to network parameters including the average shortest path length and betweenness centrality. Western blotting confirmed that CKI significantly up-regulated CASP3 expression but down-regulated the expression of MMP2, MYC and REG1A. Caspase proteins contain cysteine residues at their active site and cleave their substrate at positions next to the aspartate residue^32^. Caspase-3, a principal enzyme in the apoptotic cascade, is often used to detect apoptotic activity. Matrix metalloproteinase 2 (MMP2) has been implicated in the development and morphogenesis of tumours^33^. Increased expression of MMP2 has been shown to promote the invasion and metastasis of tumour cells^34^. Additionally, Zhao *et al*. found that the down-regulation of MYC protein in HepG2 cells significantly inhibited the migration, invasion and proliferation of HepG2 cells, suggesting that MYC might be a potential therapeutic target for HCC^35^. REG1A has been reported to be expressed in various human cancers, and it plays crucial roles in the tumourigenesis of HCC^36,37^. REG1A has also been shown to act as a factor to reduce epithelial apoptosis in inflammation^38^.

Pathway enrichment analysis uncovered the novel anti-HCC mechanisms of CKI, such as the regulation of amino acid metabolism and FRS2-mediated cascade. Metabolic disorders are involved in the pathogenesis of HCC^5,6^. Therefore, metabolomics was used to detect the differential metabolites and metabolic pathways regulated by CKI.

Metabolomics provides a valuable platform for the investigation of the metabolic perturbations in HCC cells. In the current study, ^1^H-NMR metabolomics approach was used to investigate the effects of CKI on metabolic disorders in SMMC-7721 cells. Twenty-two differential metabolites were identified after treatment with CKI, including 16 differential metabolites in cells and 10 differential metabolites in medium. Moreover, these 22 metabolites were mainly mapped to 6 metabolic pathways, which are important for identifying and analysing metabolites in biochemical reaction networks^21,39^.

Pyruvate, lactate and acetate participate in pyruvate metabolism. Tumour cells require more glucose than normal cells to support their rapid proliferation and expansion in the body^40^. Glycolysis, a universal property of malignant cells, induces acidification of the tumour environment, favouring the development of a more aggressive and invasive phenotype^41^. The Warburg effect is characterized by the capacity for using the glycolytic pathway even under aerobic conditions, indicating tumour cell-specific aerobic glycolysis^42^. Therefore, effective control of glycolytic levels is closely related to the fate of cancer cells^43,44^. The increase in the pyruvate level after the administration of CKI was possibly due to the increased rates of apoptosis, as well as the increase in aerobic glycolysis in apoptotic cancer cells^45^. The increase in lactate may be associated with increased lactate dehydrogenase activity, which is enhanced in various cancers. At the same time, the expression of lactate dehydrogenase in tumours increases, and a large amount of pyruvate is converted into lactate, resulting in an increased level of lactate^46^. In our study, after treatment with CKI, the lactate level was significantly decreased, a finding that was consistent with the previous result that antitumour drugs could decrease the lactate level^47^.

Glutamate and glutamine are involved in D**-**Glutamine and D**-**glutamate metabolism. During hepatocarcinogenesis, energy consumption grows because of cell proliferation and survival, resulting in a high uptake of glutamate^48^. Thus, glutamate levels are increased in HCC cells, whereas CKI can significantly decrease intracellular glutamate levels. The immune system is the main anti-tumour defence system in the body, and glutamine is used to maintain the basic functions of the immune system in the body. In addition, glutamine protects cells, tissues and organs from free radical damage^49^. Elevated levels of glutamine after the administration of CKI suggest that CKI may improve immune dysfunction and free radical damage.

We further reconstructed a metabolic network related to anti-HCC through MetScape based on the metabolites belonging to six metabolic pathways that were regulated by CKI. In the network, the effect of CKI on anti**-**HCC has many characteristics of multi**-**link and multi**-**level comprehensive effects. The integrative strategy presented in this study can be used as a powerful tool to understand the mechanisms of TCM.

In the current study, an integrative strategy combining the quantitative analysis of components, network analysis and experimental validation was used to explore the possible targets and pathways of CKI against HCC. After proving the efficacy of CKI on HCC, four main active compounds (matrine, oxymatrine, sophoridine and N-methylcytisine), instead of the whole ingredients of CKI were used for network pharmacology analysis. Through validating the experimental processes, the key validated targets (i.e., MMP2, MYC, CASP3 and REG1A) and key pathways (i.e., metabolism-associated pathways) were identified as mechanisms of the anti-HCC effects of CKI. Additionally, the results provide a scientific basis for the elucidation of the mechanisms of CKI against HCC.

## Materials and Methods

### Reagents and materials

CKI (total alkaloid concentration of 20.8 mg/mL) was purchased from Shanxi Zhendong Pharmaceutical Co.Ltd. (Shanxi, China), and 5-fluorouracil was ordered from Beijing Solarbio Science Technology Co. Ltd. (Beijing, China). Matrine, oxymatrine, oxysophocarpine and sophoridine were obtained from the National Institute for the Control of Pharmaceutical and Biological Products (Beijing, China; Batch numbers: 110805-200508, 110780-201508, 111652-200301, 110784-201405), and N-methylcytisine was purchased from J&K Scientific Ltd. (Beijing, China). High glucose DMEM, foetal bovine serum (FBS), 0.25% trypsin, 3-(4,5-dimethylthiazol-2-yl)-2,5-diphenyltetrazolium bromide (MTT) and dimethyl sulphoxide (DMSO) were obtained from Sangon Biotech Co. (Shanghai, China). Sodium 3-trimethlysilyl [2, 2, 3, 3-d_4_] propionate (TSP) was purchased from Cambridge Isotope Laboratories Inc. (Andover, MA, USA). D_2_O was obtained from Norell (Landisville, Pennsylvania, USA). Primary antibodies against β-actin, MMP2 and Myc were purchased from ProteinTech (Chicago, IL, USA). The caspase-3 antibody was obtained from Cell Signalling Technology, Inc. (Beverly, MA, USA). The REG1A antibody was obtained from Abcam Inc. (Cambridge, MA, USA).

### Cell culture and treatments

Human hepatoma SMMC-7721 cells were kindly donated by Professor Xiongzhi Wu (Tianjin Medical University Cancer Institute and Hospital, China). SMMC-7721 cells were maintained in DMEM culture medium supplemented with 10% FBS, 100 units/ml penicillin G, and 100 μg/ml streptomycin. All cells were cultured at 37 °C in a humidified atmosphere containing 5% CO_2_. Cells in the exponential phase of growth were used for all experiments. When SMMC-7721 cells reached 80% confluency, the cells were then continuously exposed to 1 mg/mL, 2 mg/mL, 4 mg/mL or 8 mg/mL CKI. Subsequently, the cells were exposed to 0.5 mg/mL, 1 mg/mL, or 2 mg/mL and 4 mg/mL matrine, oxymatrine, oxysophocarpine, sophoridine or N-methylcytisine, respectively.

### Cell viability assay

Cell viability of SMMC-7721 cells was evaluated using the MTT assay. Cells were seeded on 96-well plates with a density of 5 × 10^4^ cells/mL in 100 µL of medium for 24 h and then were exposed to different concentrations of agents for 24, 48 or 72 h. Following incubation, 10 μL of MTT (5 mg/ml) was added to each well. After 4 h of incubation at 37 °C, the culture medium was removed and 100 μL of dimethyl sulphoxide (DMSO, Sangon Biotech, Shanghai, China) was added to dissolve the formazan crystals. The absorbance was measured at 570 nm using a microplate reader (Infinite M200 Pro, Tecan, Switzerland), and the cell viability was expressed as a percentage of the value of the untreated group.

### Wound-Healing Assay

SMMC-7721 cells were seeded in six-well plates at a density of 5 × 10^4^ cells/mL. The centre of each well was scratched with a sterile 10-µL pipette tip. After washing with phosphate-buffered saline, different concentrations of CKI (0, 1, and 2 mg/ml) were added to the wells and then were incubated for 12, 24 or 36 h. Micrograph images were taken with a microscope at the indicated time points to observe the extent of wound closures.

### Transwell Assay

The Transwell assay was used to evaluate the migration inhibitory activities of CKI against SMMC-7721 cells. The assay was performed using a Boyden chamber with an inserted micropore membrane (6.5 mm in diameter, 8.0 μm pore size) in 24-well plates (Corning Inc., Corning, NY, USA). Next, 2 × 10^5^ cells in 200 μL of serum-free DMEM supplemented with 0.1% FBS were placed in the upper part of each chamber, whereas the lower compartments were filled with 600 μL of CKI (0, 1, and 2 mg/mL, 10% FBS). After 36 h of incubation, non-migrating cells on the top of the membrane were removed. Thereafter, the migrated cells on the bottom of the membrane were fixed, stained with 0.1% crystal violet, and observed under an inverted microscope at 100× magnification for three independent experiments.

### Sample Preparation for UHPLC

One millilitre of CKI was diluted to 10 mL with water and was filtered through a micropore membrane (0.22 mm; Jinteng Corp., Tianjin, China) before use.

For the quantitative determination of the five constituents in CKI samples, an accurately weighed amount of each reference substance was mixed and dissolved in 10 mL of methanol to obtain a stock solution with a concentration of 0.502 mg/mL for matrine, 0.494 mg/mL for oxymatrine, 0.501 mg/mL for oxysophocarpine, 0.503 mg/mL for sophoridine, and 0.499 mg/mL for N-methylcytisine.

### Ingredient identification

Ultra-performance liquid chromatography in tandem with mass spectrometry (UPLC-MS) (Thermo Fisher Scientific, Runcorn, Cheshire, UK) analysis was used to assess the main ingredients in CKI. UHPLC was conducted in tandem with mass spectrometry using a Thermo fisher U3000 UHPLC and Thermo Scientific Q Exactive mass spectrometer with an ESI source and the following parameters: mobile phase (A) acetonitrile: 0.01 mol/L ammonium acetate (pH = 8.0) = 3:2 and (B) 0.01 mol/L ammonium acetate (pH = 8.0); injection volume 5 µL; column temperature 35 °C, using a gradient elution mode. Run times were from 0 to 12 min up to 8% B and from 11 to 20 min up to 27% B. The UHPLC system consisted of an Acquity UPLC HSS T3 column (2.1 × 100 mm, 1.8 µm) (waters, USA) with a 0.3 mL/min flow rate.

### Target fishing for CKI

In our previous research^13^, HCC-related genes were collected from the liver cancer databases OncoDB.HCC^15^ (http://oncodb.hcc.ibms.sinica.edu.tw) and Liverome^16^ (http://liverome.kobic.re.kr/index.php). The validated targets of the 4 compounds were extracted from the Herbal Ingredients’ Targets (HIT) Database^50^ (http://lifecenter.sgst.cn/hit/). The predicted targets of CKI were obtained using ChemMapper^51^ (http://lilab.ecust.edu.cn/chemmapper/), an online tool for predicting targets based on 3D similarity. These targets were mapped to HCC-related genes to obtain the candidate targets of CKI.

### Network construction and analysis

The associated proteins of the targets of CKI were obtained from the String^52^ (http://string-db.org/) database. Cytoscape^53^ was applied to eliminate duplicate interactions and to construct a protein-protein interaction network. Parameters such as Average shortest path length and Betweenness centrality were calculated by NetworkAnalyzer^54^. R value was used to determine the ranks of the 48 targets by the following formula:

1 $${\rm{R}}=\frac{{X}_{i}-{X}_{i}(\min )}{{X}_{i}(\max )-{X}_{i}(\min )}\times 50 \% +\frac{\frac{1}{{X}_{j}}-\frac{1}{{X}_{j}}(\min )}{\frac{1}{{X}_{{\rm{j}}}}(\max )-\frac{1}{{X}_{j}}(\min )}\times 50 \% $$

where *X*_*i*_ is the average shortest path length, *X*_*j*_ is the betweenness centrality, and R is an indicator to evaluate the importance of a target.

### Pathway analysis

Cytoscape plugin Reactome^55^ was used to enrich the possible pathways involved in the anti-HCC effect of CKI.

### Western blot analyses

SMMC-7721 cells (5 × 10^4^ cells/mL) were seeded on 90 × 20-mm dishes. After treatment, the SMMC-7721 cells were scraped off and washed twice with cold PBS. The cells were solubilized by RIPA lysis buffer (Beyotime, China) containing 1% phenyl methylsulphonylfluoride (PMSF, Beyotime, China) for 30 min on ice. Whole-cell lysates were clarified by centrifuging at 12 000 rpm for 15 min at 4 °C, and the supernatants were collected. Protein concentrations were determined by the BCA protein assay. Equal amounts of protein (50 μg) were separated by electrophoresis on 12% sodium dodecyl sulphate polyacrylamide gels and were transferred onto PVDF membranes. These membranes were soaked in 5% skimmed milk dissolved with TBST buffer (Tris Buffer Saline supplemented with 0.1% Tween-20) for 2 h to block nonspecific binding sites. The membranes were then incubated overnight at 4 °C with the primary antibodies (MMP2^56,57^, MYC^58,59^, Caspase3 and REG1A). After washing with TBST, the membranes were incubated for 2 h at room temperature with fluorescent secondary antibodies. After rewashing with TBST, the membranes were scanned using a fluorescent scanner (Odyssey CLX, Gene Company Limited, USA).

### Cell collection for NMR analysis

All experiments included six independent replicates. Cells were harvested by scraping and then were rinsed with 4 mL of PBS after treatment with 4 mg/mL CKI for 24 h. The mixture was centrifuged at 1000 r/min for 5 min. Next, the supernatant was discarded and the cell pellet was rinsed with 4 mL of PBS. The precipitate was then collected, immediately frozen in liquid nitrogen, and stored at −80 °C. To isolate extracellular metabolites, 10 mL of extracellular medium was pipetted from cells. The samples were subsequently centrifuged at 1000 r/min for 10 min. The collected supernatant, which was used as the extracellular fraction, was immediately frozen in liquid nitrogen and stored at −80 °C.

### Sample preparation for NMR analysis

The cells and culture broth were removed from −80 °C and thawed at 4 °C according to the literature^60^ with minor adjustment. The extracellular medium was prepared for freeze-drying by taking 2 mL of the medium. Cell extraction for repeated freeze-thaw and ultrasonic disruption was conducted according to the following procedure. After repeated freeze-thawing 5 times, the cell pellets were kept on ice for 5 min before being re-suspended in 1 mL of ice-cold methanol/water (1/2, v/v), and ultrasonic disruption for 5 min on the ice (sonicate 5 s, stop 9 s). The supernatant was collected after centrifugation at 13000 r/min for 10 min at 4 °C, and 1 mL of methanol aqueous solution was added to the precipitate. The above ultrasonic sieving was repeated and the supernatant was collected two times in 5-mL EP tubes for lyophilization.

The lyophilized powder of cells and fluids of cells were dissolved in 600 µL of phosphate buffer (0.1 M, KH_2_PO_4_/Na_2_HPO_4_, pH 7.4) containing 0.005% and 0.02% TSP, respectively, as well as 10% D_2_O. After centrifugation (13,000 r/min, 4 °C, 10 min), 600 µL of supernatant was transferred into a 5-mm NMR tube for analysis.

### ^1^H-NMR Measurement

The ^1^H-NMR spectrawere recorded at 298 K using a Bruker 600-MHz AVANCE III NMR spectrometer (Bruker Biospin, Germany) and the noesygppr1d pulse sequence for water suppression. The ^1^H-NMR spectrum for each sample consisted of 64 scans requiring 5 min of acquisition time with the following parameters: spectral width 12,345.7 Hz; spectral size 65,536 points; relaxation delay of 1.0 s; acquisition time of 2.654 s. All spectra were manually phased and baseline corrected using MestReNova software (Mestrelab Research, Santiago de Compostella, Spain). Chemical shifts were referenced to TSP at δ 0.00. Regions distorted by residual water (δ 4.5~5.0) were excluded in the subsequent analysis. Each spectrum was then segmented at 0.01-ppm intervals across the chemical shift 0.50~9.00; each data point was normalized to the sum of its row and then was exported as a text file for further multivariate statistical analysis.

### Multivariate pattern recognition analysis

The normalized integral values were then subjected to multivariate data analysis using SIMCA-P 13.0 software (Umetrics, Sweden). Partial least-squares-discrimination analysis (PLS-DA) was performed to distribute and separate different groups in a supervised manner. Next, the PLS-DA model was validated by the response values of the permutation test in which the class membership was randomly shuffled 200 times. Additionally, another supervised pattern recognition approach—orthogonal projection to latent structures discriminant analysis (OPLS-DA)—was then performed to improve the classification of the different groups, as well as to screen the biomarkers. The corresponding loading, where each point represents a single NMR spectral region segment, was used to identify which spectral variables contributed to the separation of the samples on the scores plot. Variable importance in the projection (VIP) values and coefficients were also applied to screen the important variables.

### Metabolic pathway analysis

The potential metabolic pathway was analysed by using MetPA. Potential biological roles were evaluated using the MetaboAnalyst enrichment analysis tool. Metscape, the metabolic network analysis and visualization tool (http://metscape.ncibi.org./)^61^, was used to generate the compound network associated with each of the differential metabolites.

### Content determination of representative metabolites

The contents of pyruvate (PA) and glutamate (Glu) were determined according to the manufacturer’s protocols (Komin, Suzhou, China), which were based on extraction with a specific extract and then using a colour reagent for colour development.

### Construction a compound-target-metabolite network

According to the network constructed by MetScape, 174 key genes involved in differential metabolites were obtained. Subsequently, the potential interactions between the 174 genes and 48 targets of CKI were obtained from the String database. A bio-network of compound-target-metabolite was constructed using Cytoscape software.

### Statistical analysis

Quantitative data were presented as means ± standard error of mean (SEM) from three or more independent repetitions. Student’s t test was used to test the differences between two groups, and one-way ANOVA followed by Dunnett post hoc test was used for statistical analysis to determine significant differences of three or more groups. All preprocessed NMR data were imported into the software package SIMCA-P 13.0 (Umetrics, Sweden) for multivariate data analysis. P < 0.05 was deemed to indicate statistical significance.

## Electronic supplementary material

Supplementary Information
