# Supplementary material for: Uncovering the anticancer mechanism of Compound Kushen Injection against HCC by integrating quantitative analysis, network analysis and experimental validation
Source: Sci Rep. 2018 Jan 12;8:624. doi: 10.1038/s41598-017-18325-7 (PMC5766629; doi:10.1038/s41598-017-18325-7)
Supplement: Supplementary file 1 — Supplementary Information [file 41598_2017_18325_MOESM1_ESM.doc]

**Uncovering the anticancer mechanism of Compound Kushen Injection against HCC** **by integrating quantitative analysis, network analysis and experimental validation**

Li Gao,^1,*^ Ke-xin Wang,^1, 2,*^ Yu-zhi Zhou,^1^ Jian-song Fang,^3^ Xue-mei Qin^1^ **&** Guan-hua Du1, 4[[1]](#footnote-2) 


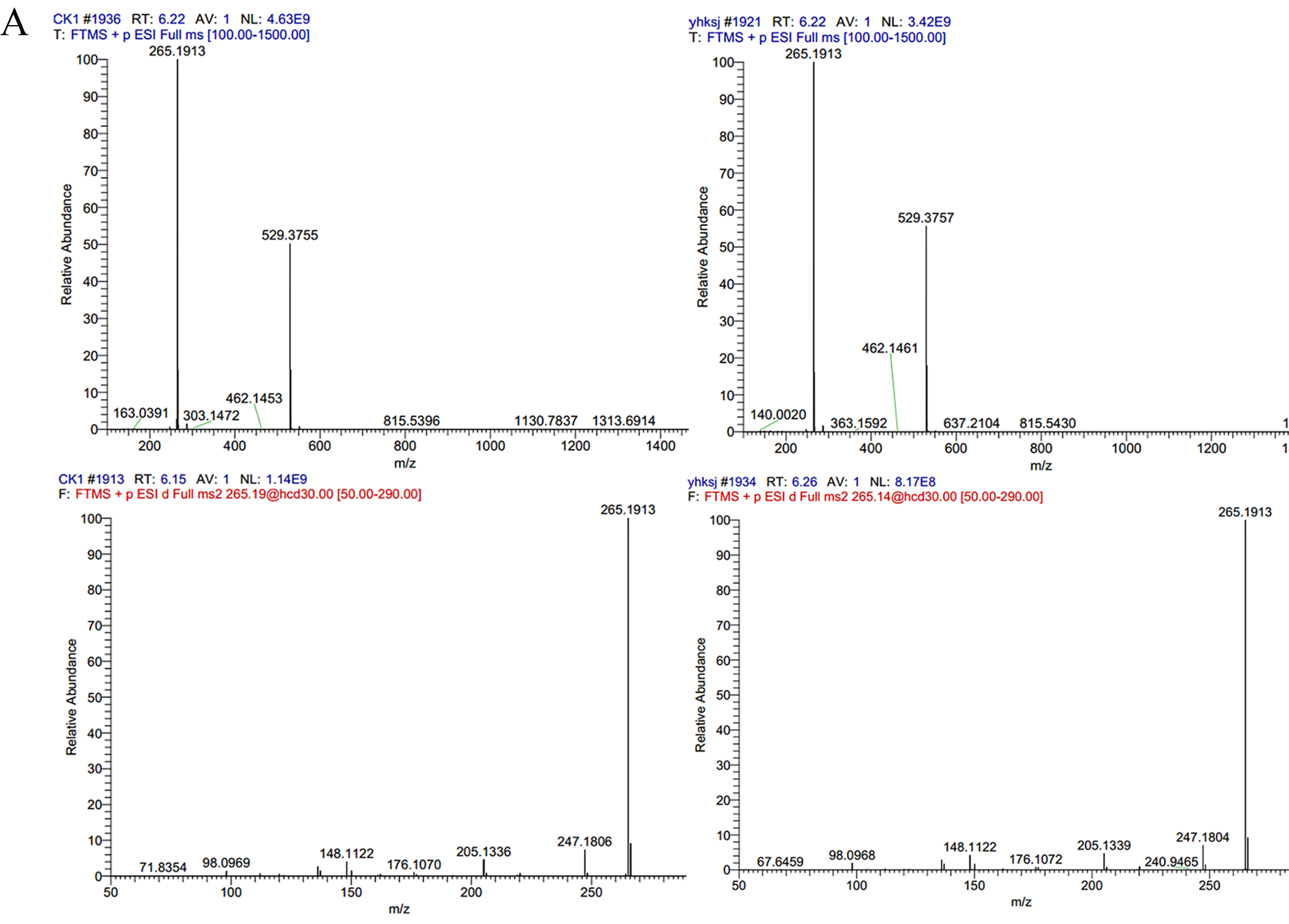


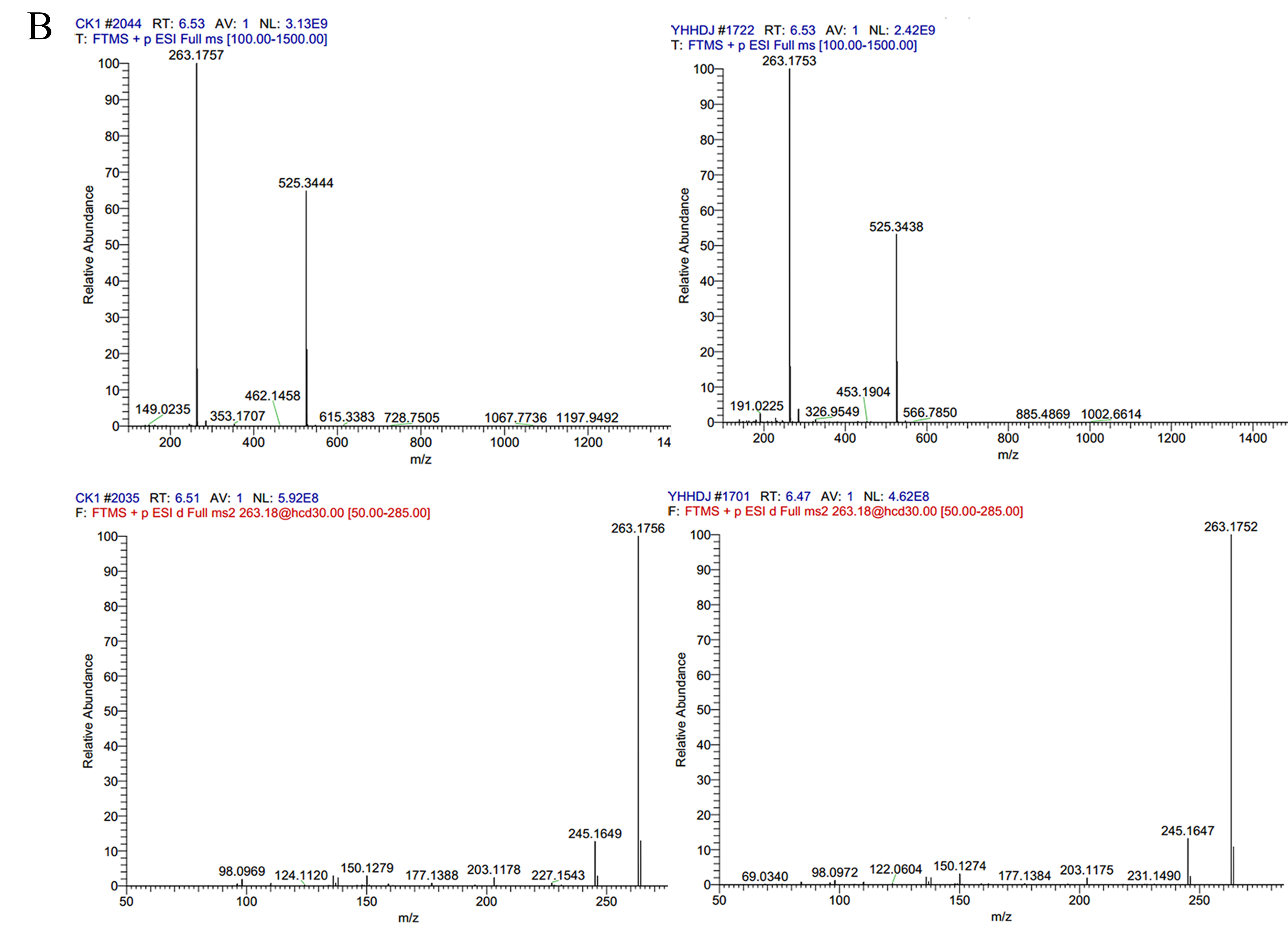


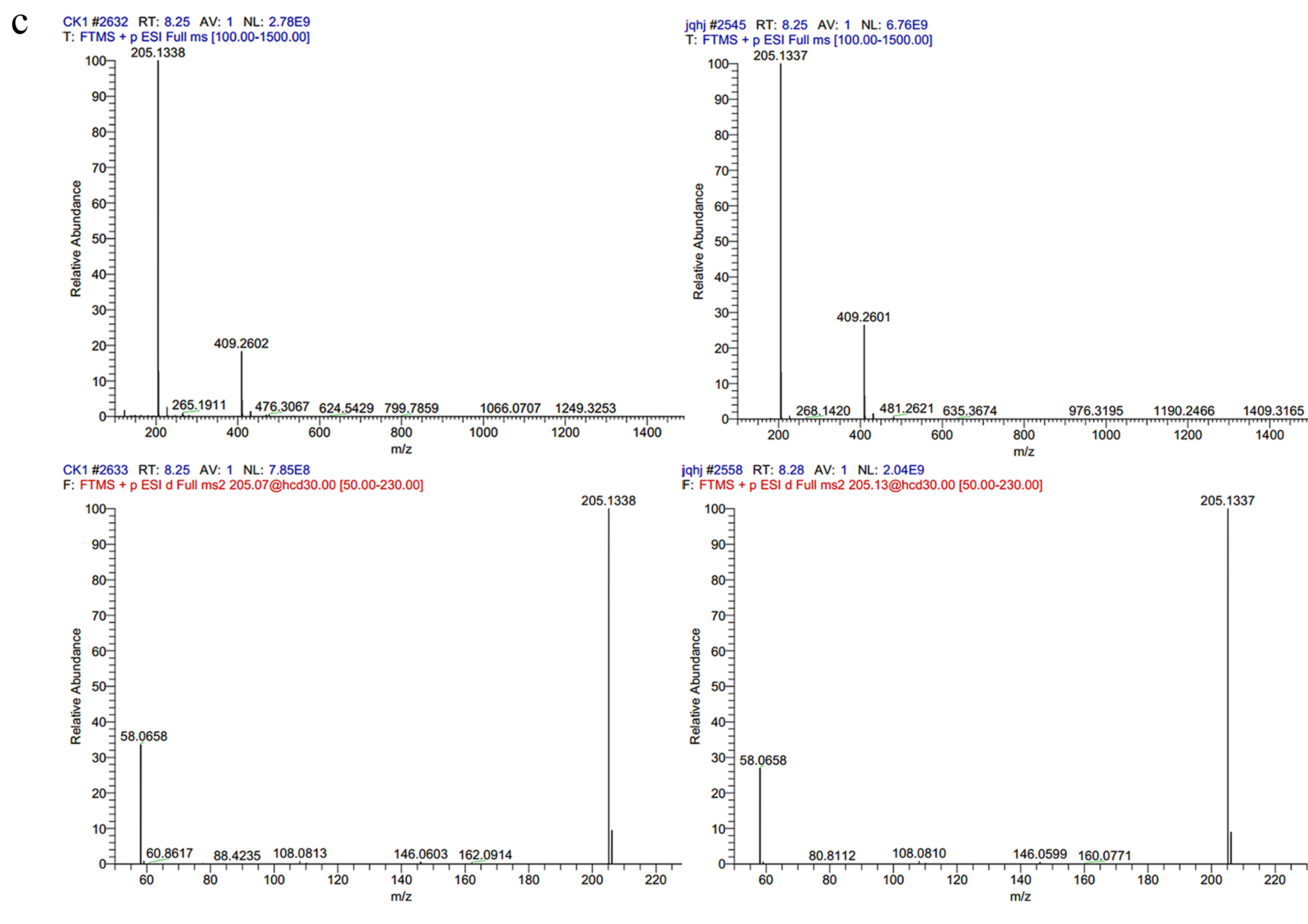


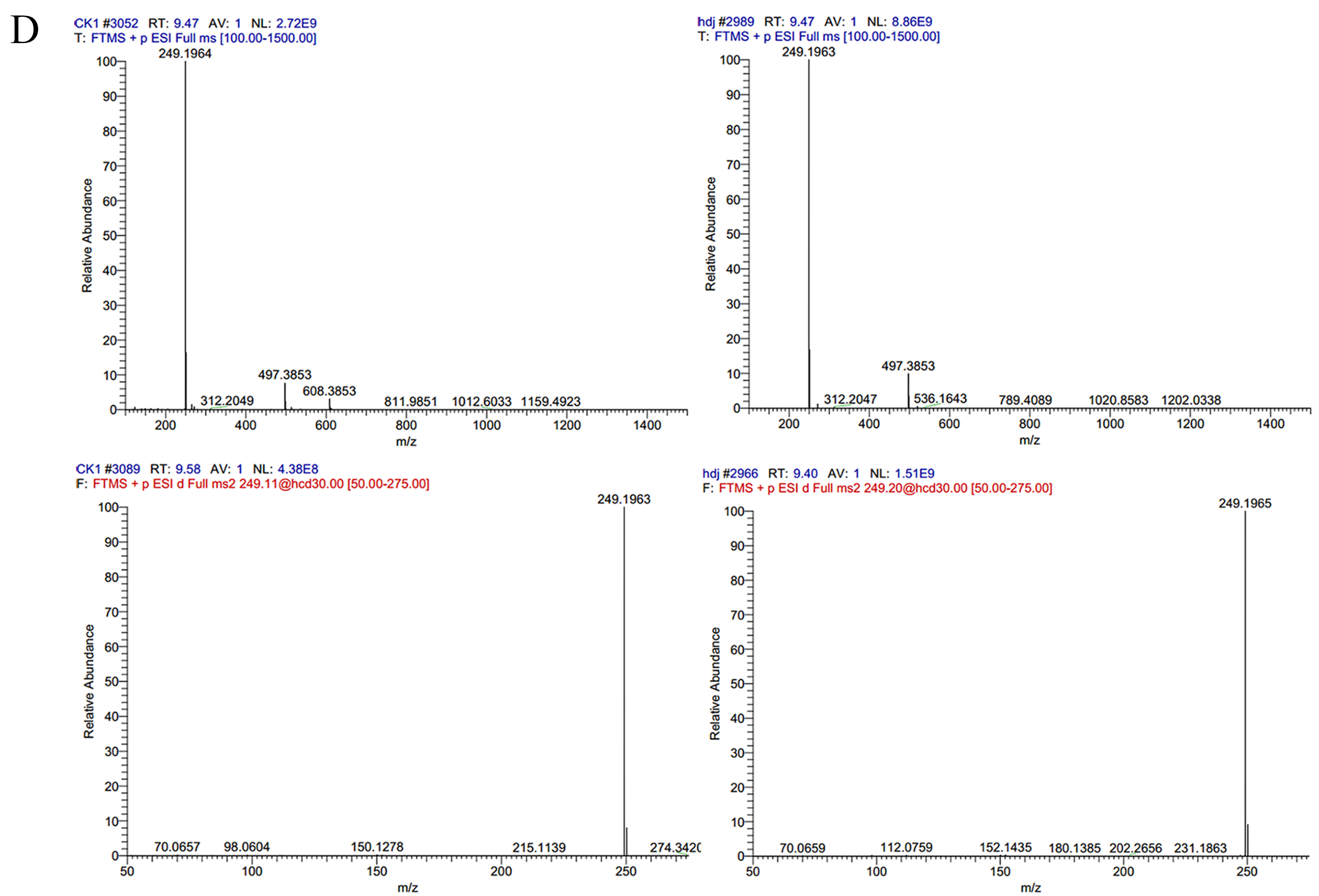


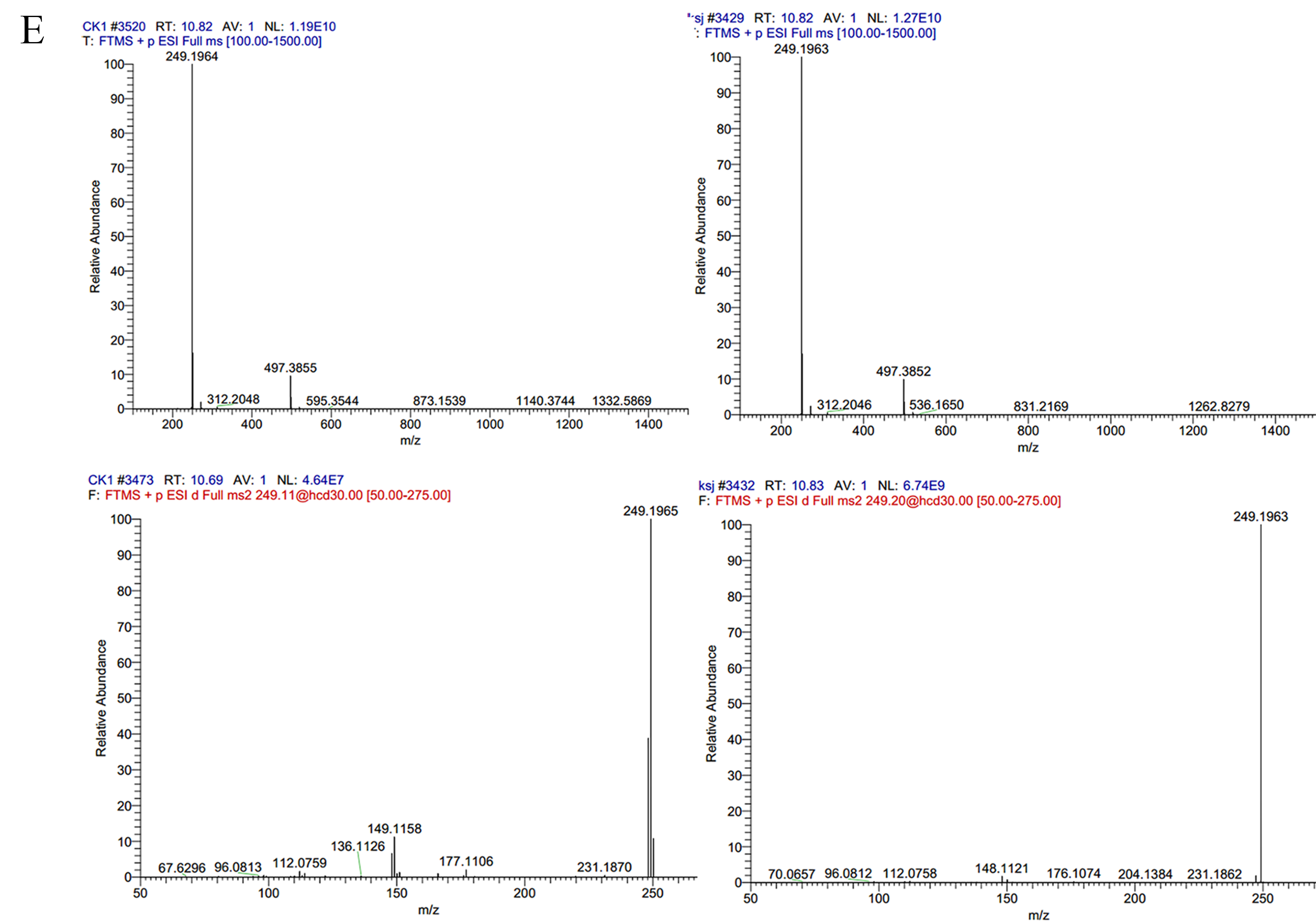


Supplementary Figure S1-S5. Mass spectra chromatograms of ingredients of CKI. A matrine, B oxymatrine, C sophoridine, D N-methylcytisine, E oxysophocarpine.


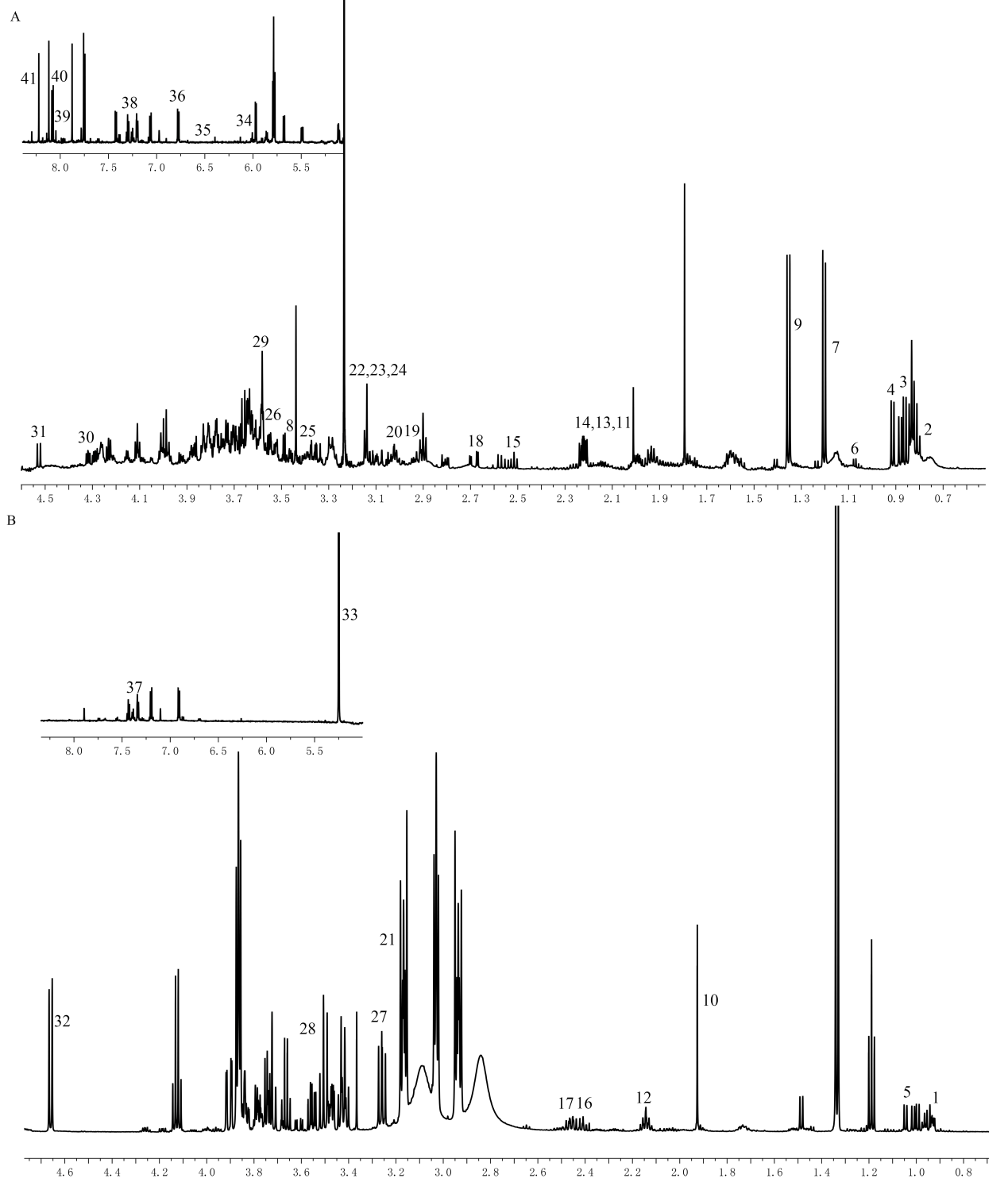


Supplementary Figure S6. Typical ^1^H-NMR spectrum of SMMC-7721 cells (A) and medium (B).


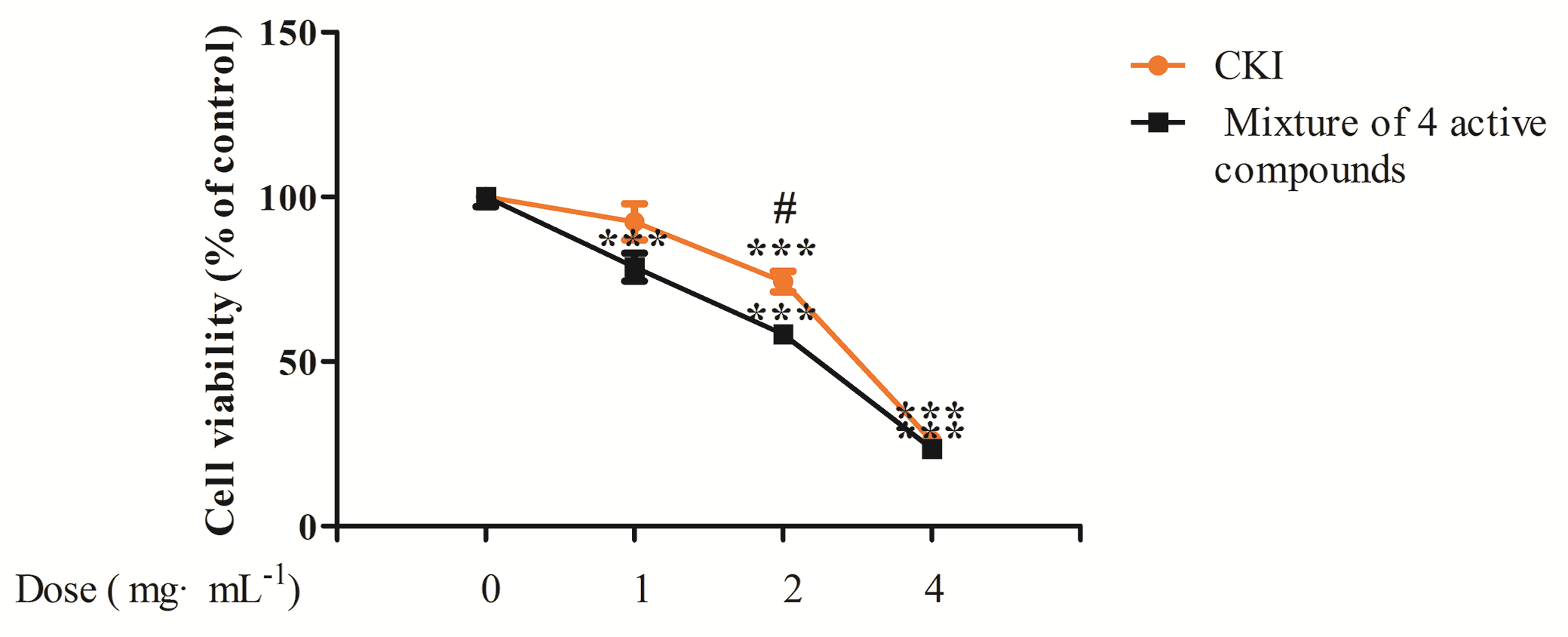


Supplementary Figure S7. Effects of CKI and mixture of 4 active compounds on proliferation of SMMC-7721 cells. The cell viabilities under different treatments were measured using MTT. Data are represented as mean ± SEM (n=6). *** *p*<0.001 versus control group. ^#^ *p*<0.05 versus mixture of 4 active compounds group.

Supplementary Table S1. ^1^H-NMR assignments of major metabolites from SMMC-7721 cells (C) and medium (M).

|  | Metabolites | Moieties | δ^1^H/(multiplicity) | Sample |
| --- | --- | --- | --- | --- |
| 1 | 2-Oxoleucine | CH_3_, CH_2_ | 0.92 (d) | M |
| 2 | isoleucine | δCH_3_, δ'CH_3_  δCH_3_, γCH_3_ | 0.93(t),1.01(d) | C, M |
| 3 | Leucine | CH=CH | 0.97(t), 0.99(d) | C, M |
| 4 | Valine | γCH_3_,γ'CH_3_ | 1.01(d),1.04(d) | C, M |
| 5 | 2-Oxovaline | CH_3_ | 1.12(d) | M |
| 6 | β-OH-butyrate | γCH_3_ | 1.18(d) | C |
| 7 | Lactate | αCH , βCH_3_ | 1.33(d), 4.12(q) | C, M |
| 8 | Threonine | γCH_3_, αCH, βCH | 1.33(d), 3.59(d), 4.26(m) | C, M |
| 9 | Alanine | βCH_3_ | 1.48(d),3.78(d) | C, M |
| 10 | Acetate | CH_3_ | 1.92(s) | C, M |
| 11 | Proline | γCH_3_,βCH_2_, αCH | 2.01 (m), 2.07 (m),4.13 (m) | C |
| 12 | Pyroglutamate | Half βCH_2_, γCH_2_, Half  βCH_2_, αCH | 2.04 (m), 2.42 (m), 2.52 (m),4.18 (dd) | M |
| 13 | Glutamate | αCH, βCH_2_, γCH_2_ | 2.09(m),2.35(m),3.78(m) | C |
| 14 | Glutamine | αCH, βCH_2_ , γCH_2_ | 2.15(m),2.46(m),3.78(m) | C |
| 15 | Glutathione | Glu β, Glu γ, Cys β, Cys α | 2.16 (m), 2.56 (m), 2.94 (m) | C |
| 16 | Pyruvate | CH_3_ | 2.38(s) | C, M |
| 17 | Succinate | CH_2_ | 2.41(s) | C, M |
| 18 | Dimethylamine | CH_3_ | 2.73 (s) | C |
| 19 | TMA c | CH_3_ | 2.91(s) | C |
| 20 | Creatine | CH_3_ , CH_2_ | 3.04(s),3.93(s) | C |
| 21 | Ethanolamine | αCH_2_, βCH_2_ | 3.15 (t), 3.86 (t) | C, M |
| 22 | choline | N(CH_3_)_3_ | 3.20(s) | C |
| 23 | PC^c^ | N(CH_3_)_3_ | 3.22(s) | C |
| 24 | GPC ^c^ | N(CH_3_)_3_ | 3.23(s) | C |
| 25 | Taurine | CH_2_SO_3_, NCH_2_ | 3.25 (t), 3.43 (t) | C |
| 26 | Myo-inositol | C5H, C1,3H, C4,6H, C2H | 3.28 (t), 3.54 (dd), 3.63 (t), 4.07 (t) | C |
| 27 | Methanol | CH_3_ | 3.36 (s) | C, M |
| 28 | Glycerol | βCH_2_, β’CH_2_ | 3.56(dd), 3.66(dd) | C, M |
| 29 | Glycine | CH_2_ | 3.56(s) | C, M |
| 30 | 1,3-Dihydroxyacetone | βCH_2_, β’CH_2_ | 3.58 (s), 4.41 (s) | C |
| 31 | Adenosine monophosphate | C5H-ribose, C4H-ribose, C3H-ribose, C1H-ribose, CH-ring, CH-ring | 4.01 (m), 4.37 (m), 4.51 (m), 6.14 (m), 8.27 (s), 8.61 (s) | C |
| 32 | β-Glucose | βC_1_H | 4.65 (d) | C, M |
| 33 | α-Glucose | CH | 5.23 (d) | C, M |
| 34 | Inosine | CH-ribose, CH-ring, CH-ring | 6.11 (d, 6.0), 8.24 (s), 8.35 (s) | C |
| 35 | fumarate | CH=CH | 6.53(s) | C |
| 36 | Tyrosine | 3 or 5-CH,2 or 6-CH | 6.91 (d), 7.19 (d) | C, M |
| 37 | Histidine | 5CH, 3CH | 7.09 (s), 7.38 (s) | M |
| 38 | Phenylalanine | 2CH, 4CH, 3CH | 7.33 (m), 7.38 (m), 7.43 (m) | C, M |
| 39 | Oxypurinol | CH | 8.18 (s) | C |
| 40 | Hypoxanthine | N-(2)CH=N, N-(7)CH=N | 8.2 (s), 8.22 (s) | C |
| 41 | Formate | CH | 8.46 (s) | C, M |

Full images of Western bots for Figure 6

1. CASP3


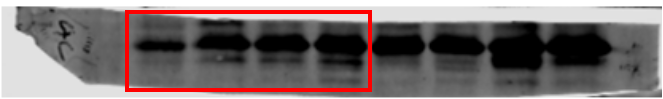


**CASP3**

**β-actin**


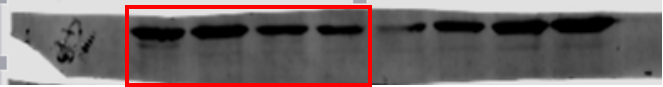


1. MMP2


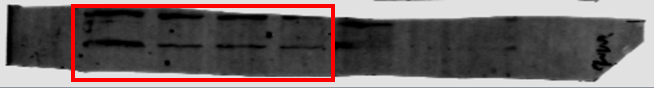


**MMP2**


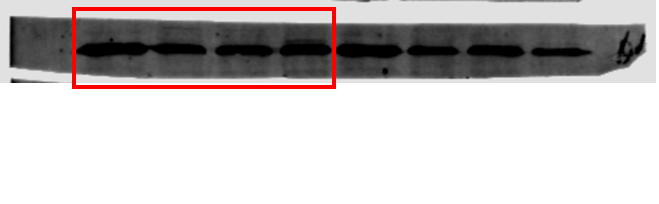


**β-actin**

1. MYC


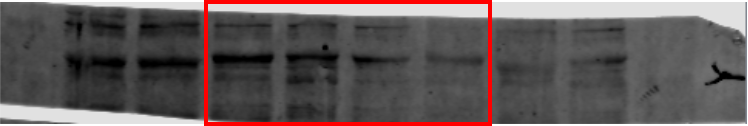


**MYC**


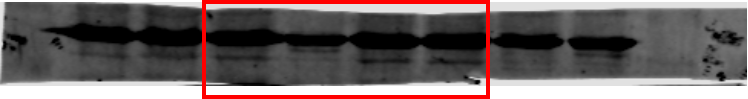


**β-actin**

1. **REG1A**


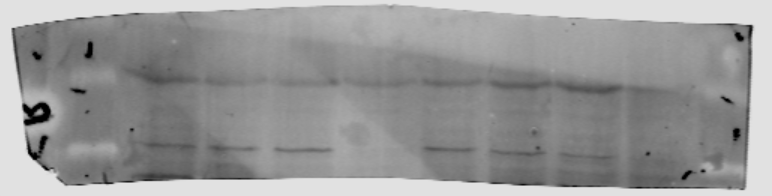


**REG1A**


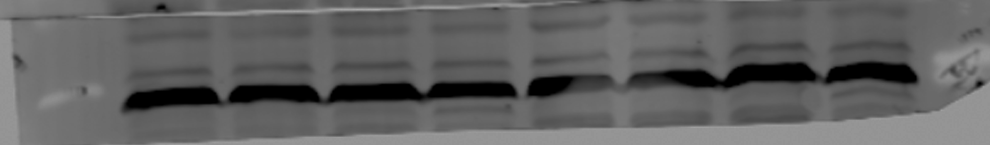


**β-actin**

Corresponding authors:

Li Gao (E-mail: gaoli87@sxu.edu.cn); Tel & Fax: 86-351-7018379; Address: No.92 Wu Cheng Road, Taiyuan 030006, China

Xue-Mei Qin (E-mail: qinxm@sxu.edu.cn); Tel & Fax: 86-351-7011501; Address: No.92 Wu Cheng Road, Taiyuan 030006, China

1. ^1^Modern Research Center for Traditional Chinese Medicine, Shanxi University, Taiyuan 030006, PR China. E-mail: gaoli87@sxu.edu.cn , Tel & Fax: 86-351-7018379; qinxm@sxu.edu.cn, Tel & Fax: +86-351-7011501

   ^2^College of Chemistry and Chemical Engineering, Shanxi University, Taiyuan 030006, China.

   ^3^Institute of Clinical Pharmacology, Guangzhou University of Chinese Medicine, Guangzhou 510405, China. ^4^Institute of Materia Medica, Chinese Academy of Medical Sciences & Peking Union Medical College, Beijing 100050, China.

   *These authors contributed equally to this work and should be considered cofirst authors. Correspondence and requests for materials should be addressed to Li Gao (gaoli87@sxu.edu.cn) and Xue-mei Qin (qinxm@sxu.edu.cn) [↑](#footnote-ref-2)
